# Mutations in SORL1 and MTHFDL1 possibly contribute to the development of Alzheimer’s disease in a multigenerational Colombian Family

**DOI:** 10.1371/journal.pone.0269955

**Published:** 2022-07-29

**Authors:** Johanna Alexandra Tejada Moreno, Andrés Villegas Lanau, Lucia Madrigal Zapata, Ana Yulied Baena Pineda, Juan Velez Hernandez, Omer Campo Nieto, Alejandro Soto Ospina, Pedronel Araque Marín, Lavanya Rishishwar, Emily T. Norris, Aroon T. Chande, I. King Jordan, Gabriel Bedoya Berrio

**Affiliations:** 1 Molecular Genetics Research Group, University of Antioquia, Medellin, Colombia; 2 Neuroscience Research Group, University of Antioquia, Medellin, Colombia; 3 Research and Innovation Group in Chemical Formulations, EIA University, Medellin, Colombia; 4 IHRC-Georgia Tech Applied Bioinformatics Laboratory, Atlanta, Georgia, United States of America; 5 PanAmerican Bioinformatics Institute, Cali, Valle del Cauca, Colombia; 6 School of Biological Sciences, Georgia Institute of Technology, Atlanta, Georgia, United States of America; Torrey Pines Institute for Molecular Studies, UNITED STATES

## Abstract

Alzheimer’s disease (AD) is the most common cause of dementia in the elderly, affecting over 50 million people worldwide in 2020 and this number will triple to 152 million by 2050. Much of the increase will be in developing countries like Colombia. In familial forms, highly penetrant mutations have been identified in three genes, APP, PSEN1, and PSEN2, supporting a role for amyloid-β peptide. In sporadic forms, more than 30 risk genes involved in the lipid metabolism, the immune system, and synaptic functioning mechanisms. We used whole-exome sequencing (WES) to evaluate a family of 97 members, spanning three generations, with a familiar AD, and without mutations in APP, PSEN1, or PSEN2. We sequenced two affected and one unaffected member with the aim of identifying genetic variants that could explain the presence of the disease in the family and the candidate variants were validated in eleven members. We also built a structural model to try to determine the effect on protein function. WES analysis identified two rare variants in SORL1 and MTHFD1L genes segregating in the family with other potential risk variants in APOE, ABCA7, and CHAT, suggesting an oligogenic inheritance. Additionally, the structural 3D models of SORL1 and MTHFD1L variants shows that these variants produce polarity changes that favor hydrophobic interactions, resulting in local structural changes that could affect the protein function and may contribute to the development of the disease in this family.

## Introduction

AD is the leading cause of dementia in Latin America. The term dementia is used to define a heterogeneous group of progressive and degenerative brain pathologies, which are clinically characterized by deterioration in memory, learning, orientation, language, comprehension, and judgment [1]. Of all dementia patients, 60 to 80 percent of cases are diagnosed with AD, which affected almost 50 million people worldwide in 2018; this number will more than triple to 152 million by 2050 [2]. Less is known about trends in low- and middle-income countries like Colombia, where the prevalence per 1000 population for dementia is 13.1 (95% CI: 8.5 to 19.3) [3] and The number of people with AD could be approximately 260.000 in 2020 but the current estimates could be underestimating 50% [4].

The hallmark pathologies of AD are the extracellular accumulation of the processing products (Aβ42) of amyloid-β protein precursor (APP), which tend to aggregate forming beta-folded sheets, (amyloid plaques), and extracellular fibrillar aggregates of the microtubule-associated protein tau (neurofibrillary tangles), which are neurotoxic [5]. Plaques and tangles are present mainly in the entorhinal cortex, hippocampus, basal forebrain, and amygdala. These brain regions are involved in learning, memory, and emotional behaviors. [6]. AD is classified considering the age of onset and the inheritance pattern, with early-onset Alzheimer’s disease (EOAD, before 65 years of age) characterized by an autosomal dominant inheritance pattern and late-onset Alzheimer’s disease (LOAD, after 65 years of age) presenting a complex inheritance pattern. Twin and family studies indicate that genetic factors play an important role in more than 80% of AD cases [7]. Heritability estimates for LOAD range from 70–80%, and EOAD shows 92 to 100% heritability [7,8]. An autosomal dominant inheritance pattern only been observed in 5% of AD families. In all other AD families the inheritance pattern is complex, and the disease is caused by a combination of both genetic and environmental factors [9,10]. Linkage studies in families provided early insights into the molecular genetics of AD. More than 350 highly penetrant mutations have been identified in three genes: *APP* [9], *PSEN1*, [11] and *PSEN2* [12] in EOAD patients [13]. But mutations in these genes are causative of the disease in only about 13% of AD patients [14]. Technological advances like genome-wide association studies (GWAS), has achieved the identification of more than 30 loci associated with AD. The APOE-ε4 allele, located in the 19q13.2 region, had been the only well-established risk factor for both EOAD and LOAD [15,16]. Collaborative efforts have changed the direction of research in the genetically complex forms of AD. At least 10 new risk loci have been identified thanks to these efforts in different genes as *CLU*, *CR1*, *PICALM* and *BIN1*, *MS4A*, *CD2AP*, *CD33*, *EPHA1*, *ABCA7 and SORL1* [17–23], implicated in pathways related to lipid processing, the immune system, inflammation, and endocytosis [20,24]. This list will probably be extended by the International Genomics of Alzheimer’s Project (IGAP) created in 2011. In the last 10 years new GWAS studies have achieved the identification of new genes associated with AD. In 2013, a meta-analysis of 74,046 individuals identifies 11 new susceptibility loci for AD (HLA-DRB5–HLA-DRB1, PTK2B, SORL1, SLC24A4-RIN3, DSG2, ZCWPW1, *CELF1, FERMT2, NME8, CASS4, MEF2C) [25]. In 2018,* Marioni, et al., reported seven additional genome-wide significant loci (ADAM10, BCKDK/KAT8, ACE, *TREML2*, *SPPL2A*, *IL-34*, PLCG2) on over 500,000 individuals from Great Britain [26]. Five new genome-wide loci (*IQCK*, *ACE*, *ADAM10*, *ADAMTS1*, and *WWOX*) and the haplotype HLA-DR15 were identified as a risk factor for LOAD by Kunlke, et al., in 2019 [27]. Finally, in 2021, four common variant loci and six rare variant loci were identified in a single-marker meta-analyses and additionally, eight new loci (TRANK1, FABP2, LARP1B, TSRM, ARAP1, STARD10, SPHK1, and SERPINB13) in gene-based analyses were reported [28].

Next-generation sequencing techniques (NGS) such as whole-exome sequencing (WES), whole-genome sequencing (WGS), and targeted sequencing (targeted-panel NGS), have expanded the possibilities to identify causal or risk variants that remain difficult to reach [29]. For instance, WES of a neurological and immunological disorders associated with cerebral autosomal dominant arteriopathy with subcortical infarcts and leukoencephalopathy (CADASIL) identified a mutation in NOTCH3 (R1231) in AD patients [30]. Later, rare variants in the *NCSTN* gene associated with LOAD were identified [31]. Through WGS, a mutation with a protective effect was identified in the gene encoding the APP protein (Ala676Thr). WGS in patients with EOAD and LOAD identified nonsense and missense mutations in *SORL1* gene [32,33]. Also, a rare susceptibility variant in *TREM2* was identified [34,35]. A target sequencing in a cohort of African descendant samples identified new AD risk variants in the ABCA7, AKAP9, COBL, MS4A6A, PTK2B, SLC10A2, and ZCWPW1 genes [36,37]. Recently, two new variants in the PDE11A gene were identified in Chinese individuals with EOAD that increase Tau phosphorylation [38].

We evaluated a multigenerational family of 97 members, spanning three generations, with an inheritance pattern suggestive of autosomal dominance from eastern Antioquia of Colombia. Eleven family members have a diagnosis of EOAD, and the index case diagnosis was verified by immunohistochemistry of the brain. In eastern Antioquia, the Neurosciences group of the University of Antioquia identify the E280A mutation in PSEN1 gene in the population under 50 years old with an autosomal dominant transmission mode [39]. We sequenced the exons of the PSNE1 gene looking for this mutation and we did not find this or any other mutation in this gene. This suggests that there must be other genetic factors involved that explain the presence of this pathology in this family. We sequenced two affected and one unaffected family members by NGS and the candidate variants found in WES were validated by Sanger sequencing in eleven family members. Finally, we build a structural model of the variants in SORL1 and MTHFDL1 genes to try to predict the effect of these variants on the function of these proteins and its role in the development of the disease in this family.

## Material and methods

### Sample size

We evaluated a multigenerational family of 97 members, spanning three generations, from eastern Antioquia of Colombia with eleven affected. We did the complete clinical and neuropsychological evaluation in five of the family members, two affected and three un affected, according the criteria of the National Institute of Neurological and Communicative Diseases and Stroke and the Alzheimer’s Disease and Related Disorders Association, NINCDS-ADRDA [40]. DNA was available from eleven family members for sequencing, four affected and seven unaffected. For the identification of variant in the PSEN1 gene, we sequence by Sanger all exons of the gen in two family members, one affected and one unaffected. Later, for the variant identification in other genes, we sequenced by NGS three family member, two affected and one unaffected. And finally, to assess the segregation of candidate variants in the family, we sequenced by Sanger the candidate variants found with WES in all eleven family members with available DNA sample. To confirm the AD diagnosis of the index case, the histological evaluation was made following the CERAD protocol stained with hematoxylin-eosin and immunohistochemistry [41] since he died and donated the brain to the Antioquia’s Neurobank

### AD diagnosis

The patients involved in the study were diagnosed with EOAD by neurologists of the Neuroscience Research Group of the University of Antioquia based on medical history and clinical, neurological, and neuropsychological examination, following the criteria of NINCDS-ADRDA [40]. To assess cognitive decline, we apply the standard cognitive tests following the Consortium to Establish a Registry for Alzheimer’s Disease (CERAD) neuropsychological test battery and additional tests validated by the Neuroscience Research Group [42]. In some cases, laboratory studies and neuroimaging were necessary for the diagnosis. For the confirmation of senile plaques, we performed a research autopsy, with brain extraction according to Neuroscience Research Group brain processing protocol to obtained the histopathological stains following the CERAD protocol stained with hematoxylin-eosin and immunohistochemistry [41] in one of the affected family members, the index case, who donated their brain to the Antioquia’s Neurobank.

### Histopathological stains

We examined 17 brain areas including Medial frontal gyrus, Superior temporal gyrus, Medial temporal gyrus, Inferior temporal gyrus, Hippocampus, Amygdala, Insula, Gyrus cinguli, Lenticular nucleus, Caudate nucleus, Thalamus, Inferior parietal lobule, Occipital lobule, Cerebellum, Mesencephalon, Pons, Medulla oblongata. 4 μm thick sections were stained with haematoxylin and eosin (H&E) further processed for immunohistochemical (IHC) staining for amyloid beta (Aβ, 1:100; BAM-10, Mob410; DBS Emergo Europe, The Hague, The Netherlands), hyperphosphorylated Tau Ser 202 and Thr 205 (tau, 1:1500; AT8, MN1020; ThermoFisher Scientific, Dreieich, Germany). Automatic immunostaining was performed with a Ventana Benchmark XT system (Roche AG, Basel, Switzerland) according to manufacturer instructions. Briefly, after dewaxing and inactivation of endogenous peroxidases (PBS/3% hydrogen peroxide), antibody specific antigen retrieval was performed, sections were blocked and afterwards incubated with the primary antibody. For detection of specific binding, the Ultra View Universal 3,3´-Diaminobenzidine (DAB) Detection Kit (Ventana, Roche) was used which contains secondary antibodies, DAB stain and counter staining reagent. Sections were scanned using a Roche–Ventana DP200 NanoZoomer automatic digital slide scanner (Roche, Germany) and obtained images of whole stained sections at a resolution of at least 1 pixel per μm.”

### Pedigree

Through direct communication with patients and their relatives, the family pedigrees were built and diagrammed using the Progeny version 7.0 (Progeny CLINICAL Version N) (Progeny Software LLC, Delray Beach, FL, www.progenygenetics.com) and Cyrillic version 3.0.400 [43] software, S1 Fig. Additional information requested from patients included personal data (age, sex, geographical origin), personal and family history of neurodegenerative diseases, clinical characteristics, neuropsychological characteristics, and support exams, among others, **S1 Table**.

### DNA samples

During the evaluation, we obtained blood samples for DNA extraction. DNA was available from eleven family members, four affected and seven unaffected. DNA was isolated from peripheral blood from four affected family members (III:1, III:5, III:9, III:10) and seven unaffected family members (III:4, III:7, III:8, IV:1, IV:5, IV:6, IV:29). The extraction of DNA was carried out following standard extraction protocols (salting out) [44], and the samples were stored at -20° C until the time of sequencing, **S1 Table**.

### E280A and PSEN1 mutations screening

Exons 4 to 13 and the flanking intron regions of the PSEN1 gene were amplified by conventional PCR. Two individuals were sequenced by bidirectional sequencing using the Sanger method, an affected individual (III:5) and a healthy relative (III:9). The chromatogram’s quality was evaluated using the program FinchTV version 1.4.0 (Geospiza, Inc.; Seattle, WA, USA; http://www.geospiza.com). For the variant detection we used Aliview version 1.18 [45] and novoSNP version 3.0.1 [46].

### Exome sequencing

The coding regions of the genome of three family members, two affected (III:5 and III:10) and one unaffected (III:7) were sequenced by NGS. This sequencing was requested by the company Macrogen in South Korea. A library enriched with the SureSelectXT Library Prep Kit was constructed, following the protocol proposed in SureSelectXT Target Enrichment System for Illumina Version B.2, April 2015. The sequencing was performed on a HiSeq 4000 device (2 x 101 base pair paired-end reads) following the protocol proposed in HiSeq 3000 4000 System User Guide Part # 15066496 Rev. HCS 3.3.52. Finally, the data was processed by the software HCS (HiSeq Control Software) version 3.3 to obtain the raw data. The data product of the sequencing was converted to the format FASTQ using the package Illumina bcl2fastq version 2.16.0.10 https://support.illumina.com/sequencing/sequencing_software/bcl2fastq-conversion-software.html.

### Bioinformatic analysis

The quality of reads was evaluated with the fastqc_v0.11.5 tool of the Babraham Institute http://www.bioinformatics.babraham.ac.uk/projects/fastqc. Then, reads were mapped against the reference human genome (UCSC hg19) using the Burrows-Wheeler Aligner tool http://bio-bwa.sourceforge.net/ [47]. Variant calling was performed following the Broad Institute’s Genome Analysis Tool Kit GATK tool (GATK) v3.8–1 Best Practices for Germline SNP & Indel Discovery in the Whole Genome and Exome Sequence https://software.broadinstitute.org/gatk/ [48]. Duplicate read removal, local sequence realignment, and base quality recalibration were performed by Picard v1.119 and GATK v3.8–1. Variants were called using GATK haplotype caller and filtered using the default criteria for variant filtration tool. Variants were annotated wANNOVAR tool of Wang Genomics Lab http://wannovar.wglab.org/ [49] and Ensembl Variant Effect Predictor (VEP) of Ensembl [50] tools. For filtering, we evaluated ~250 candidate genes compiled from Alzforum platform https://www.alzforum.org/ and used several additional variant filters following the American College of Medical Genetics and Genomics guidelines. The prioritization of the variants was carried out considering different criteria: 1. Quality of the sequences: Depth across samples (DP<30). 2. AD gene Panel from Alzforum platform 3. Type of variant: Only non-synonymous variants were considered. 4. Inheritance pattern: Autosomal dominant. 5. Allelic frequency (MAF <0.05) in a population database (1000 Genomes, ExAC, ESP6500, gnomAD) 6. Pathogenicity Predictors: variants cataloged as deleterious or possibly deleterious by more than three pathogenicity predictors including SIFT and Polyphem2 and presenting values higher than 14 by the CADD predictor.

### Clinical interpretation of candidate variants

The clinical interpretation of the genetic variants was carried out considering the guide proposed by the American College of Medical Genetics and Molecular Pathology (American College of Medical Genetics and Genomics and the Association for Molecular Pathology, ACMG Standards, and Guidelines, 2015) [51]. The Wintervar http://wintervar.wglab.org/ [52] and Varsome https://varsome.com/ [53] tools were used for the clinical interpretation of each of the identified candidate variants.

### Variants validations

To validate the variants identified in the exome analysis by NGS, each of the candidate variants were sequenced by the gold standard technique (Sanger sequencing) in the healthy and affected individuals available in the family. This service was requested by Macrogen. We sequenced the following variants: SORL1:c.C2710T:p.R904W, MAPT:c.G1667C:p.R556P, CHAT:c.G770A:p.R257Q, ABCA7:c.G2629A:p.A877T, MTHFD1L:c.G1691A:p.R564H, APOE:c.T388C:p.C130R and APOE:c.T526C:p.C176R in eleven members of the family, four affected family members (III:1, III:5 III:9, III:10) and seven non-affected family members (III:4, III:7 III:8, IV:1, IV:5, IV:6, IV:29), S11 Table.

### Structure and function of genes and proteins where candidate variants were identified

We determined the structure and function of each of the genes/proteins in which variants were identified based on information reported in different databases such as NCBI, https://www.ncbi.nlm.nih.gov/ [54] Genecards https://www.genecards.org/ [55], OMIM (Online Mendelian Inheritance in Man) https://www.omim.org/ [56], National Library of Medicine https://ghr.nlm.nih.gov/, [57], Uniprot https://www.uniprot.org/ [58], Pfam https://pfam.xfam.org/ [59], Interpro https://www.ebi.ac.uk/interpro/ [60] SMART http://smart.embl-heidelberg.de/ [61], Gene Ontology, http://geneontology.org/ [62] protein atlas https://www.proteinatlas.org/ [63] KEEG https://www.genome.jp/kegg/ [64] among others. Subsequently, a network analysis was carried out with the help of the String bioinformatics tools https://string-db.org/ [65], Mentha http://mentha.uniroma2.it/ [66], GeneMANIA http://genemania.org/ [67], IntAct https://www.ebi.ac.uk/intact/ [68], in order to determine the pathways in which these proteins would be participating and to elucidate how they could be contributing to the development of these pathology related to the nervous system.

### Protein model

A model was constructed for each of the proteins where variants were identified, for the wild type and for the corresponding variant identified in order to predict the possible effect of the variants on the structure and function of the proteins involved. For the construction of the models, different protein modeling programs were used, such as I-Tasser https://zhanglab.ccmb.med.umich.edu/I-TASSER/ [69] Swiss-Model https://swissmodel.expasy.org/ [70] Phyre2 http://www.sbg.bio.ic.ac.uk/phyre2/html/page.cgi?id=index, [71] and were visualized with Chimera https://www.cgl.ucsf.edu/chimera/ [72]. The models obtained were refined with the tools FG-MD https://zhanglab.ccmb.med.umich.edu/FG-MD/ and ModRefiner https://zhanglab.ccmb.med.umich.edu/ModRefiner/ of I-Tasser [69]. Finally, for the validation of the models, the Q-mean values of Swiss Model https://swissmodel.expasy.org/qmean/ [70] and the Ramachandran plots http://mordred.bioc.cam.ac.uk were taken into account for the selection of the best model. The models that presented the highest Q-mean values and the highest number of amino acids in the favorable region were selected according to the Ramachandran plots.

#### Ethics statement

All procedures involving experiments on human subjects are done in accord with the ethical standards of the Committee on Human Experimentation of the institution in which the experiments were done or in accord with the Helsinki Declaration of 1975. The approval of the research protocol and informed consent for the study in humans was granted by the bioethics committee of the medical research institute of the Faculty of Medicine of the University of Antioquia in the Act number 008 of May 29, 2014. Informed consent is a written document. This was read with the members of the family and after the resolution of doubts, it was signed by each individual who participated in the investigation. In the case of minors, consent was granted by signing the parents. In the case of individuals with strong neurological involvement, consent was granted by signing the responsible person. Two witnesses sign as proof of consent.

## Results

### Clinical evaluation

#### Index case (III:5)

*Clinical evaluation*. A 67-year-old patient, reports a chief complaint of short-term memory impairment (in episodic and semantic memory) and unimpaired long-term memory, with onset of symptoms at 63 years old and a slowly progressive course. Additional neuropsychiatric symptoms: insomnia, distractibility, depressive symptoms, and aggressive behavior. Two years after AD onset, he suffered a mild traumatic brain injury (TBI) that accentuated his clinical state. At 65 years old, he suffered a convulsive status epilepticus (CSE) and he was hospitalized, where they performed a cerebral and lung computed tomography (CT) showing lung cancer with cerebral metastasis. We conclude the patient had a pattern of disease compatible with EOAD, but with increased neurologic motor deterioration associated with metastatic lung cancer. More details in **S1 and S2 Appendices**.

#### Histopathological stains

**Fig 1:**
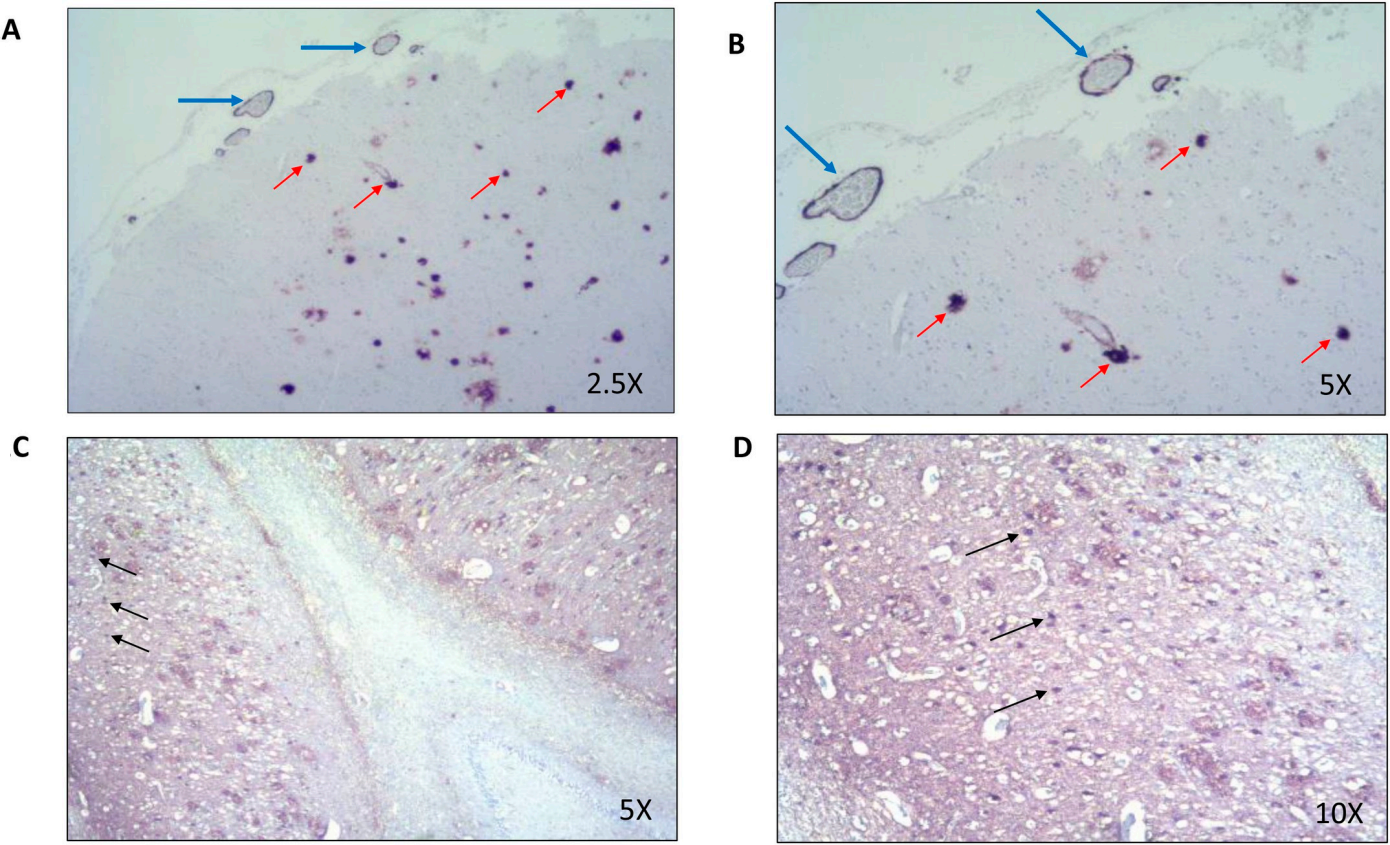
Immunohistochemistry for Alzheimer’s disease. **Fig 1A and 1B**. Immunohistochemistry for beta amyloid in the occipital cortex region. **A**. Occipital 2.5X, **B**. Occipital 5X. Multiple neuritic plaques are observed (red arrows), as well as amylodean angiopathy (blue arrows), which affects the meningeal vessels. **Fig 1C and 1D**. Immunohistochemistry for tau protein in the hippocampus region at the level of the lateral geniculate body. **C**. Hippocampus CGL 5X, **D**. Hippocampus CGL 10X. Multiple tangles and neurofibrillary threads are observed(black arrows), that affect the different portions of the hippocampus.

Conventional morphological study, staining reactions and immunohistochemistry, shows the presence of classic neuritic plaques with dystrophic and pathological neurites in the isocortex cortex/frontal association (up to >20/mm2), in the temporal isocortex (up to 17 mm2) and in the parietal isocortex (up to 19 mm2) according to a plate score correlated by age CERAD C [41]. Immunohistochemical evidence of diffuse deposits of positive β-amyloid in the frontal neocortex, central gray matter (Entorhinal region) as well as in the CA4 region according to phase 4 described by Thal, D.R., et al. (2002) [73]. Also, a scant detection of β-amyloid in vascular walls, that involves vessels in the entire circumference, and occasionally vessels in a few regions with circumferential β-amyloid affecting parenchyma and meninges, corresponding to a score of 1 in CAA, according to Love, et al. (2014) [74]. **Fig 1A and 1B**. In the staining reactions as well as in the immunohistochemistry for the tau protein, the trans entorhinal, entorhinal, pyramidal (CA1-CA4) layer, the dentate gyrus, striatum, and thalamus are affected, with involvement of the neocortex. And intraneuronal tangles and extracellular neurofibrillary threads stage V are seen according to Braak and Braak (1991) [5]. **Fig 1C and 1D**. No Lewy bodies were observed in the different structures, when stained with α-synuclein, corresponding to a stage 0 [75]. Absence of atherosclerosis of small arterial blood vessels in central gray matter and centrum semiovale without significantly affected perivascular neuropil. Amylaceous bodies are observed in a moderate amount in the hippocampus. In the frontal cortex (middle frontal gyrus) and hippocampus, infiltrating lesion is observed that destroys the morphology of the cortex and the white matter; composed of cuboid and highly mitotic cells. When studying tumor tissue, highly vascularized, infiltrating tissue is observed, with abundance of mitotic cells, ovoid, fusiform, small, and hyperchromatic nucleus, compatible with metastatic lung tumor.

### Genetic evaluation

#### Extended genealogy

We constructed a genealogy of five generations with 97 individuals 11 of which were affected. The mode of inheritance was suggestive of autosomal dominant transmission. We observed affected individuals in all generations, with a proportion of men and women affected proportionally in the first three generations. Since AD is a disease that develops mainly in old age, individuals of the last two generations (20–38 years old individuals) were asymptomatic. **S1 Fig**.

#### PSEN1 gene

No mutations were observed in any of the exons of the *PSEN1* gene analyzed in the family with AD. The family members evaluated in this study are not carriers of E280A mutation, the most prevalent mutation in Antioquia’s population [76] The polymorphism rs165932 located in intron 8 of the PSEN1 gene was identified. Both the affected individual (III:5) and the unaffected individual (III:7) carried the variant, being heterozygous G/T. **S2 and S3 Figs**. The rs165932 polymorphism is considered benign according to the VEP tool (Variant Effect Predictor) of Ensembl. https://www.ensembl.org/Tools/VEP, **Table 1**.

**Table 1:** Analysis of *rs165932* polymorphism using VEP (Variant Effect Predictor) tool version 1.18.

Variation	Location	Allele	Consequence	IMPACT	SYMBOL	Existing variation	Allele Frequency	CLIN_SIG
rs165932	14:73198145–73198145	T	Intron variant	MODIFIER	PSEN1	rs165932	0.6715	benign

**Variation:** SNP identifier **Location:** Position in hg19/CHR37 reference genome. **Allele:** Reference allele. **Consequence:** Variant type (coding variant, intron variant, regulatory region variant, etc.). **IMPACT:** A subjective classification of the severity of the variant consequence, based on agreement with variant annotation tools (e.g., snpEff). **SYMBOL:** Gene symbol. **Existing Variation:** Identifier co-located known variants. Allele Frequency: Allele frequency in 1000 genomes data base (all populations). **CLIN_SIG:** Clinical significance according ClinVar database.

#### Exome sequencing

WES revealed 71,854 variants in total, with 53,063 variants per individual on average. After applying the filters, an average of 41,834 SNPs (single nucleotide polymorphisms) and 3,364 INDELs (Insertion/Deletion) were identified characterized per individual, **S2 Table**. Of these variants, 45% are in intronic regions, 14% upstream, and 10% downstream of the gene regions. The remaining 55% correspond to variants located in coding regions; of these, 70% were classified as non-synonymous, of these, 59% correspond to missense variants, 5% to frameshift variants, and 2% to stop-loss or stop gain variants. **S4 Fig**.

#### Variant detection

No mutations in the *APP*, *PSEN1* and *PSEN2* genes were found. Nine variants found in other genes did meet the criteria for candidate variants: c.C2710T, p.R904W in SORL1 (Sortilin related receptor 1); c.G1667C, p.R556P in MAPT (Microtubule-associated protein tau); c.G770A, p.R257Q in CHAT (Choline O-acetyltransferase); c.5302delC, p.L1768fs, c.G643A, p.G215S and c.G643A, p.G215S in ABCA7 (ATP binding cassette subfamily A member); c.A5673G, p.I1891M in LPA (Lipoprotein A), c.G1691A; p.R564H in MTHFD1L ((Methylenetetrahydrofolate dehydrogenase (NADP+ dependent) 1 like)), and c.T388C, p.C130R in APOE (Apolipoprotein E), **Tables 2 and S3–S6**.

**Table 2:** Description of candidate variants under the prioritization criteria identified with the ANNOVAR tool in AD family.

Chr	Position	Ref	Alt	Gene	AA Change	dbSNP	1000G	1000G CLM	ExAC	gnomAD	Geno Canyon	fitCons	GERP ++RS	SIFT	Poly phen2 HDIV	Poly phen2 HVAR	CADD	III:7 Health	III:10 Aff	III:5 Aff
Chr11	121429346	C	T	SORL1	exon20 C2710T R904W	rs148966249	0.0002	**0.0053**	4.119e-05	1.219e-05	1.000	0.722	3.65	**D**	**D**	**D**	**33**	0/0	0/0	0/1
Chr17	44073870	G	C	MAPT	exon6 G1667C R556P	.	.	.	.	.	0.996	0.581	5.62	**D**	**D**	**D**	**24.5**	0/0	0/1	0/0
Chr10	50854563	G	A	CHAT	exon8 G770A R257Q	rs201616704	0.0002	0.0053	0.0003	0.0003	1.0	0.497	5.16	**D**	**P**	**B**	**25.4**	0/0	0/0	0/1
Chr19	1058841	C	-	ABCA7	exon39 5302delC L1768fs	.	.	.	.	4.532e-06	.	.	.	.	.	.	.	0/1	0/0	0/1
Chr19	1043103	G	A	ABCA7	exon8 G643A G215S	rs72973581	0.02	0.0160	0.0432	0.0424	1.000	0.651	-5.86	T	B	B	0.004	0/0	0/0	0/1
Chr19	1050996	G	A	ABCA7	exon19 G2629A A877T	rs74176364	0.023	0.1011	0.0169	0.0207	1.000	0.696	2.59	**D**	**B**	**B**	**19.47**	0/1	0/0	0/1
Chr6	160961137	T	C	LPA	exon37 A5673G I1891M	rs3798220	0.051	0.1915	0.0448	.	0.003	0.487	2.99	T	D	D	16.65	0/0	0/1	0/1
Chr6	151270231	G	A	MTHFD1L	exon16 G1691A R564H	rs61748674	0.0062	**0.0160**	0.0127	.	1.000	0.719	5.9	**D**	**D**	**D**	**31**	0/0	0/1	0/0
Chr19	45411941	T	C	APOE	exon4 T388C C130R	rs429358	0.15	0.1543	0.1843	.	0.194	0.635	3.02	T	B	B	0.007	0/1	1/1	0/1

**Chr:** Chromosome. **Start:** Variant start position. **Position:** Position in hg19/CHR37 reference genome. **Ref:** Reference allele. **Alt:** Alternate allele. **Gene:** Gene name. **AA Change:** Amino acid change. **dbSNP:** Variant identifier in dbSNP database. **1000G:** Allele frequency in 1000 genomes data base (all populations). **1000GCLM:** Allele frequency in 1000 genomes data base (Colombian population). **ExACFreq:** Allele frequency in ExAC 65000 data base (all populations). **gnomAD:** Allele frequency in gnomAD database, exome data (all populations). **GenoCanyon:** Conservation scores with GenoCanyon tool (Conserved region = scores~1). **fitCons:** Conservation scores with fitCons tool: (Conserved region = ~1). **GERP++RS:** Conservation scores with GERP++RS tool (Conservation region = scores>4.4). **SIFT:** Pathogenicity prediction with SIFT tool: D = Deleterious, T = Tolerated). **Polyphen2HDIV:** Pathogenicity prediction with PolyPhem2 tool for Mendelian disease variants (D = Damaging, P = Possibly Damaging, B = Benign, U = Unknown). **Polyphen2HVAR:** Pathogenicity prediction with PolyPhem2 tool for all human disease-causing mutations (D = Damaging, P = Possibly Damaging, B = Benign, U = Unknown). **III:7:** Non-affected family member. **III:10:** affected family member. **III:5:** Affected family member. **Genotype**: 0 = Reference allele, 1 = Alternate allele.

#### Clinical interpretation of candidate variants

According to the results of the Varsome and Intervar platforms, three of the candidate variants, SORL1:c.C2710T: p.R904W, CHAT:c.G1124A:p.R375Q and MTHFD1L:c.G1691A:p.R564H were classified as PP3 (pathogenic supported) because multiple lines of computational evidence like DANN, GERP, dbNSFP, FATHMM, MetaLR, MetaSVM, Mutation Assessor, Mutation Taster, PROVEAN, SIFT and/or LRT, among them, support a deleterious effect on the gene or gene product. The MAPT variant c.G1667C:p.R556P was classified as PM2 (moderate pathogenic) because it is absent from controls (or at extremely low frequency if recessive) in exome or genome sequencing projects such as 1000 Genomes, ExAC or gnomAD. Finally, the APOE variant c.T388C:p.C130R is classified as BA1 (Benign only), BP4 (Moderate Benign), PM1 (Moderate pathogenic), PP5 (Pathogenic supported) and PS3 (Pathogenic strong) simultaneously due to its high frequency (>0.05) in genome or exome sequencing projects and multiple lines of computational evidence suggesting that there is no impact on the gene or genetic product, this variant is located in a region with a high mutation rate (hot spot) and/or located in a well-established functional domain (for example, an enzyme’s active site), Uniprot identifies this variant as associated with a disease (Hyperlipoproteinemia 3) and also well-established *in vitro* or *in vivo* functional studies supportive of a damaging effect on the gene or gene product. Finally, ABCA7 variant c.G2629A:p.A877T was classified as VUS (Variant with Uncertain Significance) because no rule has met the criteria, **Table 3**.

**Table 3:** Clinical interpretation of the candidate variants identified in the exome analysis in a family with Familial Alzheimer’s Disease.

Chr	Gene	Change	dbSNP	F1III:7	FIII:10	F1III:5	Varsome	Intervar
chr11	SORL1	exon20:c.C2710T:p.R904W	rs148966249	0/0	0/0	0/1	PP3	PM1, PP3
chr17	MAPT	Exon11:c.G1667C:p.R556P	.	0/0	0/1	0/0	PM2	PM2
chr10	CHAT	exon8:c.G1124A:p.R375Q	rs201616704	0/0	0/0	0/1	PP3	PM2, PP3
chr19	ABCA7	exon39:c.5302delC:p.L1768fs	.	0/1	0/0	0/1	BP4	-
chr19	ABCA7	exon8:c.G643A:p.G215S	rs72973581	0/0	0/0	0/1	BP4	BS1, BP1, BP4
chr19	ABCA7	exon19:c.G2629A:p.A877T	rs74176364	0/1	0/0	0/1	VUS	PM1, BS1, BP1
chr6	LPA	exon37:c.A5673G:p.I1891M	rs3798220	0/0	0/1	0/1	BA1, BP4	PM1, BA1, BS1, BS2
chr6	MTHFD1L	exon16:c.G1691A:p.R564H	rs61748674	0/0	0/1	0/0	PP3	PM1, BS1
chr19	APOE	exon4:c.T388C:p.C130R	rs429358	0/1	1/1	0/1	BA1, BP4, PM1, PP5	PS3

**Chr:** Chromosome. **Gene**: Name of the gene. **Change:** Nucleotide/amino acid change. **dbSNP:** Identifier of the variant in the dbSNP database. **Varsome:** Classification of variants according to the Varsome platform. **Intervar:** Classification of the variants according to the Intervar platform. **BA1:** Benign alone. **BP1:** Benign supported. **BP4:** Benign supported. **BS1:** Benign Strong **BS2:** Benign Strong. **PM1:** Pathogenic Moderate. **PM2:** Pathogenic Moderate. **PP3:** Pathogenic supported. **PP5:** Pathogenic supported. **PS3:** Pathogenic Strong. **PS4:** Pathogenic strong. **VUS:** Variant with Uncertain Significance. **III:7:** Non-affected family member. **III:10:** Affected family member. **III:5:** Affected family member.

#### Validation and segregation analysis of genetic variants

**Fig 2:**
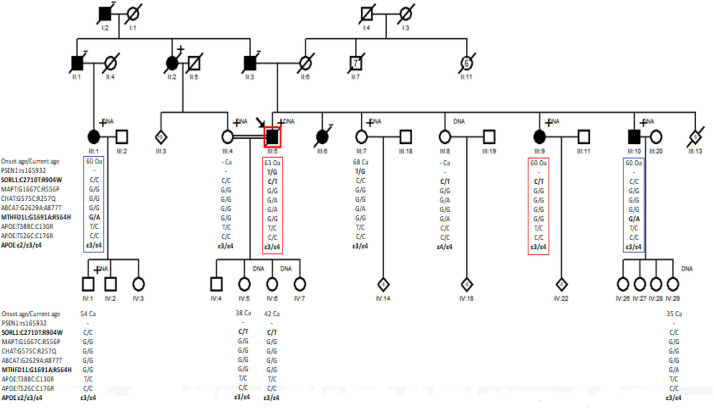
Reduce pedigree of AD family with SORL1:c.C2710T:p.R904W, MAPT:c.G1667C:p.R556P, CHAT:c.G770A:p.R257Q, ABCA7:c.G2629A:p.A877T, MTHFD1L:c.G1691A:p.R564H, APOE:c.T388C:p.C130R and APOE:c.T526C:p.C176R variants suggesting an oligogenic inheritance. **Squares**: Males. **Circles**: Female. **Filled symbols**: Affected family members. **Empty symbols**: Non-affected family members. **DNA**: Indicates family members with DNA samples. **+ symbol**: Indicate family members with neurological evaluation. **Oa**: Onset age. **Ca**: Current age. **Red dotted line**: Affected family members carrying the R904W variant in SORL1 gene. **Blue dotted line**: Affected family members carrying the R564H variant in MTHFD1L gene.

Fig 2 shows the results of Sanger sequencing of the PSEN1:rs165932, SORL1:C2710T:R904W, MAPT:G1667C:R556P, CHAT:G575C:R257Q, ABCA7:G2629A:A877T, MTHFD1L:G1691A:R564, APOE:T388C:C130R and APOE:T526C:C176R variants, and the APOE haplotype ε2/ε3/ε4 in a EOAD family. Both the affected individual (III:5) and the unaffected individual (III:7) are G/T heterozygotes for the rs165932 variant of the PSEN1 gene. The SORL1:C2710T:R904W variant was identified in two of the affected members (III:5 and III:9) and three of the unaffected members (III:8, IV:5, and IV:6) of the family. The MAPT:G1667C:R556P variant initially identified in the exome analysis in the affected individual (III:10) could not be identified by Sanger sequencing in any of the family members. The CHAT:G575C:R257Q variant was identified in the index case (III:5), and one of the unaffected members (III:8) of the family. The ABCA7 variant: G2629A:A877T was identified in the index case (III:5) and none of the unaffected family members. The MTHFD1L: G1691A: R564H variant was identified in two of the affected members (III:1 and III:10) and two of the unaffected members (III:8 and IV:29) of the family. Four of the affected members (III: 1, III: 5, III: 9 and III: 10) and six of the unaffected members (III: 4, III: 7, IV: 1, IV: 5, IV: 6 and IV: 29) of the family carry the ε3/ε4 haplotype and only one of the unaffected members (III: 8) carries the ε4/ε4 haplotype for APOE.

The c.C2710T, p.R904W variant in SORL1 gene and the c.G1691A, p.R564H variant in MTHFD1L gene do not segregate in all affected family members and are present in some unaffected cases. The c.C2710T, p.R904W variant in SORL1 gene is present in two affected family members: 1. in the index case (III:5) with an onset of 63 years and age of death of 68 and, 2. in one of the sisters (III:9) with an onset age of 60 years and a current age of 70 years at the time of the study. And the c.G1691A, p.R564H variant in MTHFD1L gene is present in another two affected family members: 1. in another sister (III:1) with an onset of 60 years and current age of 68 and, 2. in one of the brothers with an onset age of 62 years and a current age of 66 years at the time of study. The sister of the index case (III: 8) who carries the variant c.C2710T, p.R904W variant in SORL1 gene was evaluated at the age of 60 years and was considered healthy because at the time of the study she did not manifest memory complaints. The other two carriers of these variants are in the fourth generation and are daughters of the index case. The first daughter (IV: 5) had a current age of 38 years and the second daughter (IV: 6) was a current age of 42 at the time of evaluation and they were considered healthy because they were too young to start the cognitive deterioration process. It is important to note that patients in this family began their cognitive decline after 60 years of age, Fig 2. None of the risk alleles we found were homozygous. Most of the individuals in the family are heterozygous for the ApoE4 haplotype. All individuals in the family are carriers of at least one ApoE-Ɛ4 allele. AD risk is increased 3–5 fold for heterozygous APOE-ε4. Also, the affected family members (III:5 and III:9), in addition to carrying one ApoE-Ɛ4 allele, carry the variant c.C2710T in SORL1 gene in a heterozygous state. And the affected family members (III:1 and III:10) in addition to being heterozygous for the ApoE-Ɛ4 allele are heterozygous for the variant c.G1691A, in MTHFD1L gene. All healthy family members are carriers of at least one ApoE-Ɛ4 allele, but they are not carriers of the variants in the genes SORL1 and MTHFD1L. Except for the individuals (III:7 and IV:5 and IV:6) who at the time of the evaluation were too young to identify symptoms of the disease, Fig 2.

#### Function and structure of genes and proteins where candidate variants were identified

The function and structure of the genes and proteins where candidate variants were identified in the family with AD are found in the **S7 and S8 Tables**. The network constructed by the STRING tool of SOL1L protein is made up of 11 nodes and 27 vertices, has an average degree of nodes of 4.91 and an average local grouping coefficient of: 0.86. This tool yields evidence of interaction of the SORL1 with APP (Amyloid-β protein precursor), VPS35 (Vacuolar protein sorting-associated protein 35), APOE (Apolipoprotein E), BIN1 (Myc box-dependent-interacting protein 1), CLU (Clusterin), VPS26A (Vacuolar protein sorting-associated protein 26A), PICALM (Phosphatidylinositol-binding clathrin assembly protein), VSP29 (Vacuolar protein sorting-associated protein 29), FURIN (Furin) and GAA1 (ADP-ribosylation factor-binding protein) proteins. These data support the interaction between SORL1 and APP. The evidence comes from expression studies, laboratory experiments, databases, and data mining. It is important to note that five proteins with evidence of interaction are involved in the processing and clearance pathway of amyloid-beta peptides and have previously been associated with Alzheimer’s disease, including the SORL1 protein, **S5A Fig**.

#### Structural model of SORL1 protein

**Fig 3:**
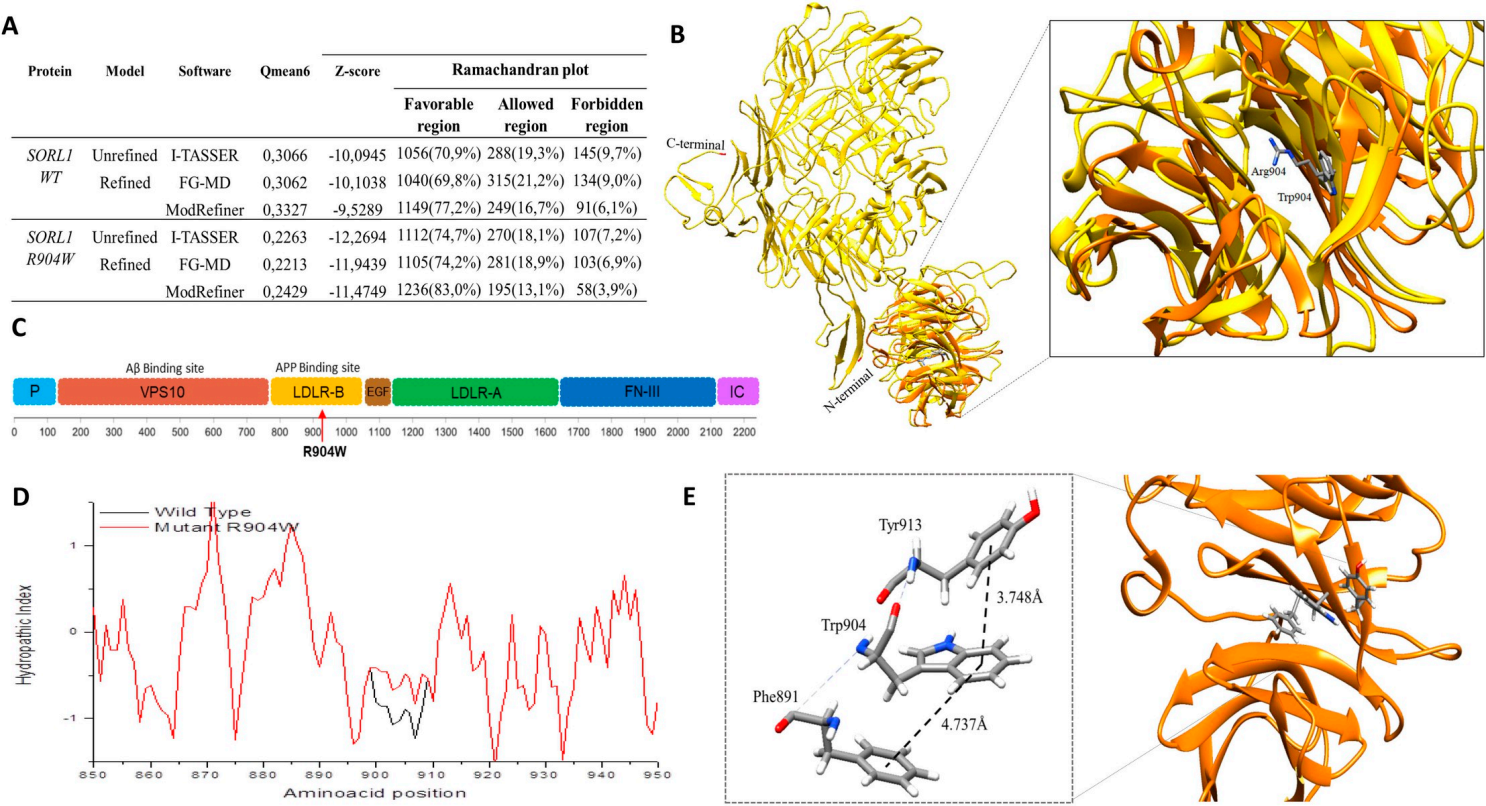
SORL1 structural model. A. Energy and stereochemistry validation of structural models of SORL1 protein. **B**. Structural model of SORL1 protein with UCSF Chimera software and detail of amino acid change R904W of the SORL1 protein. **C**. Domains of SORL1 protein. Modified from E. Louwersheimer et al. P: Pro-peptide. VPS10: Vacuolar protein sorting domain 10. LDLR-B: LDL-receptor class B repeats. EGF: Epidermal growth factor precursor type repeat. LDLR-A: LDL-receptor class A repeats. FN-III: Fibronectin type-III repeats. IC: Intracellular component. LDL: Low density lipoprotein. **D**. Hydropathic index. Black: Wild type, Arginine at position 904 of the SORL1 protein. Red.: R904W, Tryptophan at position 904 of the SORL1 protein. **E**. Co-planarity interaction of the amino acids Phe (phenylalanine 92) -Trp (Tryptophan 105) -Tyr (Tyrosine 114).

In RCSB Protein Data Bank database [77], the structure of the sortilin-1 receptor (SorLA) is not completely resolved and only one region has been crystallized by diffraction of X-rays with a resolution of 2.35Å. We use 3WSX, 3WSY and 3WSZ as a template for model building [78]. The structure was completed using a structural prediction method using the protein sequence stored on the Uniprot platform (ID: Q92673) [79,80]. The hypothetical structural model for the receptor was obtained with I-Tasser, [81–83] and with Phyre2 tool [71]. The best model for wild type receptor, (QMEAN6 = 0.3327 and Z-score = -9.5289) and SorLA-R904W (QMEAN6 = 0.3327 and Z-score = -9.5289) was obtained with the I-Tasser tool, and this model was refined with Model Refiner tool, **Fig 3A and 3B and S6 and S9 Tables**. Ramachandran plot shows that 72% of amino acids for the wild type SorLA model are in the favorable zone, 16.7% are in the allowed zone and 6.1% in the forbidden zone, and for the SorLA-R904W, 83.0% of amino acids are in the favorable zone, 13.1% are in the allowed zone and 3.9% in the forbidden zone, **Fig 3A and S7 and S9 Tables**. Hydropathic Index analysis of the SorLA protein showed that the amino acid change, Arg904Trp, produces a variation in the hydrophobicity and hydrophilicity values scores in the flanking region (from amino acid 900 to amino acid 910) according to Kyte and Dolittle coefficients calculated with Protscale software from the Swiss ExPASY suite [84], **Fig 3D**. The hydrophobicity increases cause SorLA to fold within itself when gaining non-covalent intramolecular forces, from the β-antiparallel sheets that with their aromatic residues in the distance of accessible links of 3,748 Å and 4,737 Å, favor alternate stacking interactions between specific amino acids in the side chain, which explains the structural change from the chemical point of view and the possible alteration of protein function, **Fig 3E**.

#### Structural model of MTHFD1L protein

**Fig 4:**
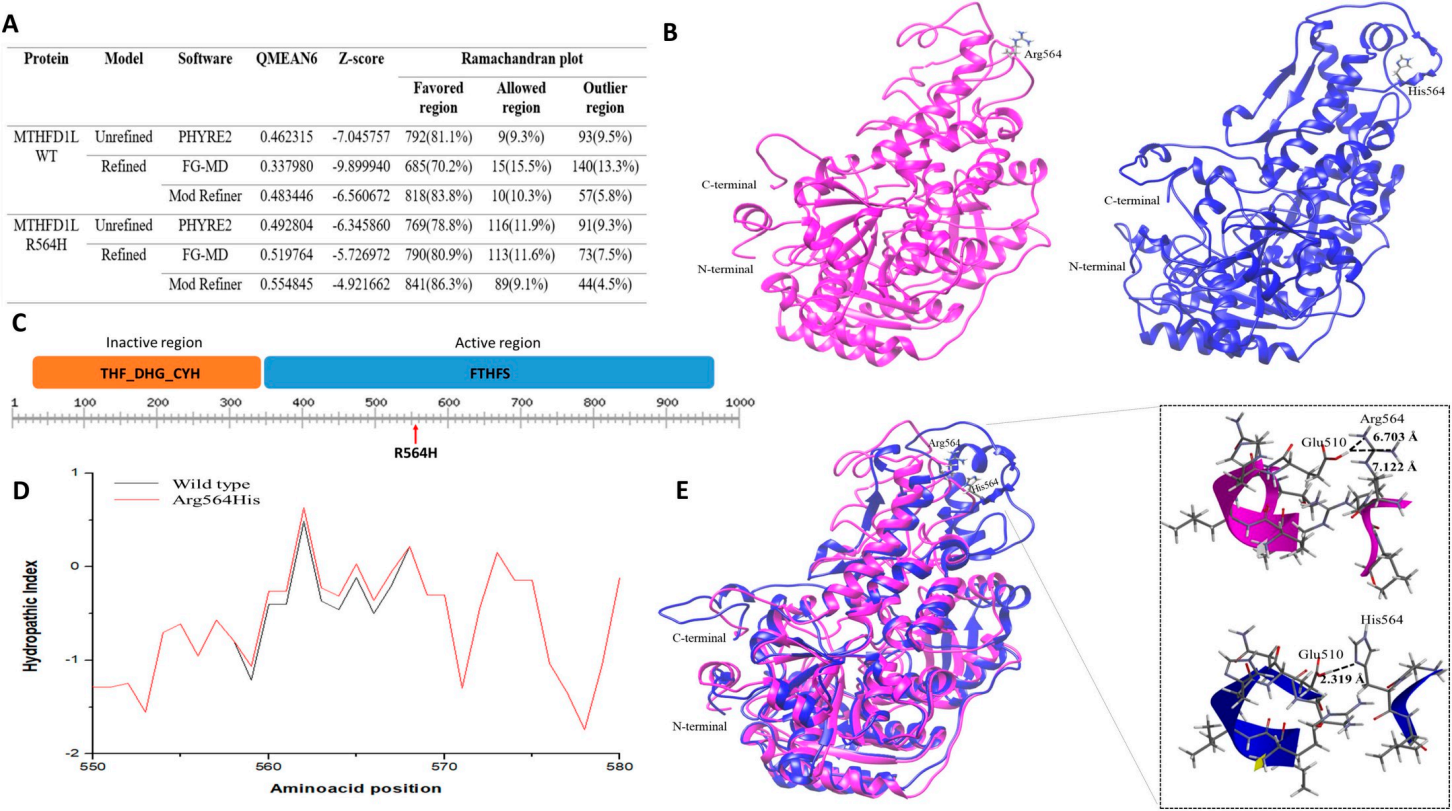
MTHFD1L structural model. **A**. Energy and stereochemistry validation of structural models of MTHFD1L protein. **B**. Structural model of MTHFD1L protein with UCSF Chimera software: Hypothetical model of the wild type of enzyme (magenta) and Arg564His variant (Blue). **C**. Domains of MTHFD1L. THF_DHG_CYH: N-terminal inactive methylene-THF dehydrogenase and cyclohydrolase domain. FTHFS: Active larger formyl-THF synthetase C-terminal domain. **D**. Hydropathic index. Black: Wild type, Arginine at position 564 of the MTHFD1L protein. Red.: R564H, Histidine at position 564 of the MTHFD1L protein. **E**. 3D alignment of the MTHFD1L enzyme: Hypothetical model of the wild type of enzyme (magenta) and Arg564His variant (Blue). Three-dimensional representation of the fragments in secondary structure for ribbons and sticks: Above (magenta) fragment for the wild type of enzyme at position Arg564; Bottom (Blue) fragment for the His564 variant.

This enzyme only has a structural fragment crystallized by solid nuclear magnetic resonance (NMR), with an ID of 2EO2 as recorded in RCSB Protein Data Bank database (ID:2EO2) [77]. Therefore, we generate prediction models using I-Tasser [81–83] and Phyre2 tools tool [71], using the sequence reported in the Uniprot database (ID:Q6UB35) [79,80]. The best structural model for wild type enzyme (QMEAN6 = 0.483446 and Z-score = -6.560672) and MTHFD1L-R564H (QMEAN6 = 0.483446 and Z-score = -6.560672) was built with Phyre2 tool, and these models were refined with the Model Refiner tool, **Fig 4A and 4B and S8 and S10 Tables**. 83.8% of amino acids are in the favorable zone, 10.3% are in the allowed zone and 5.8% in the forbidden zone for the wild type MTHFD1L, and 86.3% of amino acids are in the favorable zone, 9.1% are in the allowed zone and 4.5% in the forbidden zone for the MTHFD1L-R564H, according to Ramachandran plots, **Fig 4A and S9 and S10 Tables**. Hydropathic Index analysis [84] showed an increase in hydrophobicity from the amino acid 558 to amino acid 568, due to change from a charged polar amino acid for a weakly charged amino acid, **Fig 4D**. The 3D model of wild type and MTHFD1L-R564H shows an evident topological difference in the region adjacent to the variant with the histidine less exposed. Additionally, the minimum energy values show values of 2441.98 kJ/mol and 2089.95 kJ/mol for the wild type and the carrying variant protein, respectively, which means that the structure of the wild type is more stable. Finally, although the change from Arginine to Histidine does not alter the nature of the basic functional group, the basic contribution of the imidazole group of Histidine is shorter in binding distance than the contribution of the di-amino of Arginine, generating an acid-base interaction between Glutamic acid and Histidine with a distance of 2,319Å, suitable for a formation of adduct by hydrogen bonds that produces a structural approach, possibly altering the functional domain of the protein, **Fig 4E**.

## Discussion

The extended family from Antioquia, Colombia studied here has approximately 97 individuals with eleven affected family members. The age of onset of these patients was between 55 and 65 years, so they were diagnosed with EOAD. We found a brain with high-grade neurodegenerative changes in the form of 1. Diffuse deposits of positive B-amyloid, neurofibrillary tangles and neuritic plaques with a score of A3, B3 and C2. According to the current National Institute for Classification of Aging, this results in a "high change in neuropathology of AD considered as a sufficient explanation for dementia" [85], 2. The evaluation of amyloid angiopathy shows limited involvement of vessels, with scant involvement of meningeal and parenchymal vessels, indicating a very low vascular compromise associated with AD [74], 3. Diffuse infiltration of small, highly mitotic cells that affect the morphology of the cortex and white matter that correspond to metastatic lesions of lung cancer [86]. In accordance with the current guidelines of the National Institute on Aging, the clinically known dementia of the patient is adequately explained by the "neuropathological changes of AD".

No previously reported variants in PSEN1 were found, included the E280A mutation, the most prevalent mutation in the Antioquia’s population [76]. We identified the *rs165932* polymorphism located in intron 8 PSEN1 gene. The rs165932 polymorphism was initially reported as a risk factor for LOAD with an OR = 1.97, 95% CI 1.29–3.00. In this case, the T allele in the homozygous state confers a double risk for LOAD [87]. Although these findings were subsequently replicated [88–91], there is controversy because studies in other populations have not found an association of this intronic variant with AD [92–96]. Other studies have associated the G allele with a protective effect [97,98]. However, an inverse effect has been found in other studies [99,100] where the G allele has been identified as a risk factor for AD. This polymorphism has also been associated with EOAD [97,99,101] with similar results to those reported for LOAD. The affected and healthy individuals sequenced are both heterozygous for the T allele. Since these data are inconclusive to determine an association of the T allele with AD in these families, we decided to broaden the search for variants in other genes that may explain the development of the disease in the studied family.

The index case carries variants in SORL1, CHAT, ABCA7, LPA, and APOE genes. SORL1 encodes the Sortilin-related receptor, SorLA. This protein is a multifunctional endocytic receptor that is widely expressed in the central nervous system in particular in the cerebellum, cerebral cortex, hippocampus and in the caudate nucleus, is involved in the uptake of lipoproteins and proteases and participates in APP traffic to and from the Golgi apparatus. Therefore, it probably acts as a classification receptor that protects APP from late endosome traffic and its processing to beta-amyloid peptide, thus reducing the formation load of amyloidogenic peptides.

Its structure consists of several domains with different functions: an N-terminal domain VPS10 (vacuolar protein sorting domain) important for the classification and transport of endosomal proteins and can also interact with different neuropeptides, participate in the processing of APP, 5 LDL domains- Class B receptor (Low-density lipoprotein-YWTD domain receptor) that play a central role in cholesterol metabolism, an epidermal growth factor type domain, which plays a vital role in immune responses, as well as in the elimination of dead cells in the organism, and, epidermal growth factor precursor type repeat (EGF), 11 class A LDL-receptor domains, identified as the lipoprotein binding site and 6 type III fibronectin domains, involved in cell adhesion processes, cell morphology, thrombosis, cell migration and embryonic differentiation and a fibronectin type III domains (FNIII), **Fig 3C**. SorLA interacts (via N-terminal ectodomain) with APP, forming a 1:1 stoichiometric complex, this interaction retains APP in the trans-Golgi network and reduces processing into soluble APP- and amyloid-beta peptides [102–105]. The R904W variant is in the extracellular region of the protein, specifically in the third LDL-receptor class B domain that extends from amino acid 888 to 932, important in cholesterol metabolism and APP binding site.

Both common variants with modest OR values and rare missense, stop codon and protein-truncating variants, (PVT) in the SORL1 gene have been associated with AD, both in familial and sporadic forms in different populations [106–113]. The use of NGS techniques has contributed to the identification of variants associated with AD in this gene [32,114–119], including the Alzheimer’s Disease Sequencing Project (ADSP), which performed a complete sequencing of the 5,740 patients with late-onset Alzheimer’s disease and 5,096 cognitively normal controls, mainly of European descent, which includes 218 cases and 177 controls of Hispanic origin, in which SORL1 has been one of the most prevalent genes [120]. However, the exact functional consequences of most of the rare variants identified, as well as their corresponding levels of risk for the development of AD are yet to be determined. Only the effect of some of these variants, PVT, that generate a loss of protein function have been studied *in vitro* showing variable degrees of decreased protein function, leading to an increase in the secretion of beta-amyloid peptide [106,121,122].

The mechanism by which the variants in the receptor, SorLA, may be associated with AD are the following: 1) APP binds through the LDL domain and can redirect it to the non-amyloidogenic pathway, inhibiting the formation of beta-amyloid peptide, and 2) binds to nascent beta-amyloid peptides and directs them to the lysosome, preventing their secretion [104,106,121,123–125]. Functional studies of the p.Gly511Arg variant showed an interrupted interaction of the Vps10p domain with monomers of the beta-amyloid peptide, which reduced the lysosomal orientation of the beta-amyloid peptide by SORL1 [105]. Regarding the p.Glu270Lys and p.Thr947Met variants, both showed an increase in the secretion of the Aβ1–40 and Aβ1–42 forms of the amyloid-beta peptide and the levels of APP on the cell surface in transfected cell lines [118]. The analysis of the structural model of Arg904Trp variant shows that the polarity changes in the flanking region can affect the protein structure possibly affecting its function, its interaction with the membrane and even blocking its functional domain.

CHAT encodes the enzyme Choline O-acetyltransferase, ChAT, whose expression in the CNS is characteristic of cholinergic neurons given its function since it is responsible for the synthesis of the neurotransmitter acetylcholine. Its structure consists of a colin/carnitine acetyltransferase domain, which participates in the transfer of an acyl group from one compound (donor) to another (acceptor). The G1124A variant is found in exon 8 of the gene and the R375Q change is located within the colin/carnitine acetyltransferase domain that extends from amino acid 131 to 719 and about 145 amino acids from the Coenzyme A binding site that covers 13 amino acids of the 520 to 532 of the enzyme. A moderate number of variants in ChAT associated with AD have been reported, among the most supported are rs3810950, rs2177369, rs1880676 and rs868750. The rs3810950 polymorphism has been shown to be associated with AD in more than a dozen studies in different populations of Asia, America, and Europe mainly [126–138]. These variants can affect the synthesis of the enzyme, amplifying a cholinergic neurotransmission deficit in Alzheimer’s disease [126–138]. The association of these variants in ChAT is also related to the response to AChEI therapy [139,140].

The affected sibling (III:10) of the index case does not share the variants identified in the index case, however this individual is a carrier of the variant c.G1691A:p.R564H in the MTHFD1L. The protein encoded by MTHFD1L, a Methylenetetrahydrofolate Dehydrogenase (NADP+ Dependent) 1 Like protein is involved in folate metabolism, specifically, in the synthesis of tetrahydrofolate (THF) in the mitochondria. THF is important in the *de novo* synthesis of purines and thymidylate and the regeneration of methionine from homocysteine. This monofunctional enzyme consists of two main domains: an inactive N-terminal methylene-THF dehydrogenase and cyclohydrolase domain from amino acid 31 to 348 and an active C-terminal formyl-THF synthetase (FTHFS) domain of amino acid 349 to 978. The G1691A variant is found in exon 16 of the gene and the R564H change is located in the C-terminal domain of the active formyl-THF synthetase (FTHFS), **Fig 4C**. Elevated plasma homocysteine levels have been linked to AD [141,142] and other neurodegenerative diseases, including Parkinson’s disease [143], and have been recognized as a risk factor for preeclampsia [144], diabetic complications [145], heart disease [146–148] and coronary artery disease (CAD) [149].

The mechanisms by which the association between folate metabolism and AD can be explained are the following: 1. Folate is a cofactor in the metabolism of carbon, during which it promotes the remethylation of homocysteine, a cytotoxic amino acid that contains Sulfur that can induce DNA chain breakage and oxidative stress, promoting the generation of reactive oxygen species (ROS) and cell death by apoptosis [150–152] and 2. Elevated homocysteine contributes to the risk of AD by causing vascular alterations, which have been directly related to AD and can cause a cholinergic deficit in cortical neurons due to its toxicity [153]. This gene has also been associated with neural tube defects that include spina bifida, meningocele, encephalocele, and anencephaly, as a result of abnormalities in proliferation, differentiation, and death of neural cells [154–156]; and with adenocarcinoma and it has been considered as a new molecular target for cancer therapy [157–160]. The R564H variant found in the exome analysis was evaluated in the structural model that was built. This variant produces an increase in hydrophobicity in the adjacent region and produces a change in the topology of the protein generated by the possible formation of an adduct, which can finally alter the functional domain of the protein and therefore its function, **Fig 4E**.

The ABCA7 gene encodes the ATP Binding Cassette Subfamily A Member 7. This protein is responsible for transport of various molecules through extracellular and intracellular membranes, playing an important role in the homeostasis of lipids and macrophage-mediated phagocytosis. This transporter has been predominantly detected in myelo-lymphatic tissues with greater expression in peripheral leukocytes, thymus, spleen, and bone marrow, and although it is also expressed in the CNS where it participates in the clearance of beta-amyloid peptide by microglia and macrophage cells, limiting the production of beta-amyloid by playing a role in the regulation of endocytosis and/or APP processing. Its structure consists of two highly conserved ATP-binding domains (ATPase domain), the first (ABC transporter 1) located from amino acid 807 to 1038 and the second (ABC transporter 2) located from amino acid 1793–2025, which use the energy product of the hydrolysis of ATP for the export or import of a wide variety of substrates ranging from small ions to macromolecules. The G2629A variant is found in exon 19 of the gene and the A877T change is in the first ATP binding domain important for ATP hydrolysis.

Finally, the variant rs429358: c.T388C: p.C130R in the APOE gene was identified in the several affected individuals of the family. This gene encodes apolipoprotein E involved in lipid metabolism. The rs429358: c.T388C: p.C130R variant in the APOE gene is classified as a strong pathogenic (PS3) since well-established *in vitro* or *in vivo* functional studies support a harmful effect on the gene or the gene product and pathogenic supported (PP5) since reliable sources recently remark the variant as pathogenic. This variant together with the variant rs7412: c.C526T: p.R176C located in exon 4 of the APOE gene constitutes the haplotype APOE (derived from the combination of rs429358 and rs7412). APOE-Ɛ4 is the most important genetic risk factor for AD, and this risk increases according to the number of copies of the allele. Heterozygous individuals, carriers of one copy of the ApoE-Ɛ4 allele have twice the risk, while homozygous individuals carrying two copies of the ApoE-Ɛ4 allele have eleven times the risk of developing the disease in relation to those carrying the other ApoE alleles ApoE-Ɛ3 and ApoE-Ɛ2. The ApoE-Ɛ3 allele, the most common in the population, is considered neutral, while the ApoE-Ɛ2 allele is considered protective, this being the least frequent in the population [161–166]. All family members have almost one copy of ApoE-Ɛ4 allele, which raises the risk of developing AD twice, according to the literature [161–166].

Although the implication of many of these genes as risk factors is highly discussed, when the susceptibility genes belong to the same signaling pathway, the risk associated with a multigenic disease can be better explained by relating the possible integrated effects of the variants in the genes that intervene in the same pathway rather than with the individual effect of each of the variants in a single gene separately [167,168]. Recent studies have reported similar results to those we found in this study. 1. WES revealed no mutations in the *PSEN1*, *PSEN2*, and *APP* genes in any of the family members. 2. WES detected possible pathogenic rare variants segregating in multigenerational families with autosomal dominant transmission like in the *SORL1*, *ABCA7*, and *APOE* genes. 3. The *SORL1* variants were present in both affected family members and some of the unaffected family members, and, in some cases, affected non-carries were reported, raising interrogations on the inheritance pattern, or suggesting incomplete penetrance, **Fig 2**. 4. The variants are in a highly conserved amino acid, affecting an important functional domain, has a CADD score higher than 14, and was predicted to be deleterious for more than three pathogenicity predictors [169,170].

As limitations of the study, we have a small sample size since it was not possible for us to take biological samples from all affected and unaffected individuals of the family to perform genetic analyzes, even though the multigenerational family is very large. Some members of the family live in rural areas with difficult access, and it was not possible to carry out the home visit. This also limited the performance of additional tests to refine the diagnosis of all individuals in the family, since some did not have the possibility of traveling to the city for these tests. Only individuals who had a complete clinical evaluation were included in the study. Finally, it is important to be clear that although the WES is an effective tool for identifying genetic variants in families with Mendelian inheritance patterns, this type of study only covers 2% of the whole genome, therefore, 98% of non-coding genome remains unexplored. It is possible that genetic variants located in this region (introns, splicing regions, regulatory regions) contribute to the development of neurological diseases, and considering that epigenetic mechanisms also play a role in mediating synaptic and neural network connectivity and plasticity, epigenetic mechanisms can also be involved in the molecular pathophysiology of these diseases. However, it was possible to carry out the clinical evaluation of a considerable number of individuals of the family in three generations, and the confirmation of the diagnosis of AD in the index case, who donated the brain for the identification of amyloid plaques and neurofibrillary tangles in the histopathological examination. And it was possible to identify variants previously associated with AD such as (APOE:c.T388C:p.C130R), as well as new variants in genes previously associated with AD such as SORL1:c.C2710T:p.R904W and MTHFD1L:c.G1691A:p.R564H. “Nevertheless, additional studies are required to determine how these changes could affect the protein function and if these changes could be contributing to the development of the disease in this family. This may be prospects for future studies.

## Conclusions

We found possibly pathogenic genetic variants in SORL1 and *MTHFD1L* genes and other risk variants in *CHAT*, *ABCA7*, *and APOE* genes segregating in a Colombian multigenerational family with EOAD, suggesting an oligogenic model where multiple genetic factors may be interacting in different biological pathways related with the Aβ production and/or clearance, contributing to the risk of AD. 0020 build for the variants R904W in SORL1 and R564H in MTHFDL1 shows that these changes may produce polarity variations that favors hydrophobic interactions, resulting in local structural changes that could affect the protein function and may contribute to the development of the disease in this family. Additional studies are required to determine how these changes could affect the protein function and if these changes could be contributing to the development of the disease in this family.

## Supporting information

S1 Fig Extended Pedigree of family with five generations. (PDF)

S2 Fig Results obtained from the analysis of PSEN1 gene sequences in the family members affected with AD using Alivew software version 1.18. (PDF)

S3 Fig Results obtained from the analysis of the sequences in the family members affected with AD using novoSNP software version 3.0.1. (PDF)

S4 Fig Classification of the genetic variants found in Family with AD using VEP tool. (PDF)

S5 Fig Protein-protein interaction analysis using STRING tool. (PDF)

S6 Fig Structural Model of SORL1 protein. (PDF)

S7 Fig Ramachandran plot of SORL1 protein. (PDF)

S8 Fig Structural Model of MTHFD1L protein. (PDF)

S9 Fig Ramachandran plot of MTHFD1L protein. (PDF)

S1 Table Evaluations carried out on members of family with familial Alzheimer’s disease. (PDF)

S2 Table Number of variants identified in the of variant calling and hard filtering process with the GATK program in a family with AD. (PDF)

S3 Table Description of candidate variants under the prioritization criteria identified with the ANNOVAR tool in AD family. (PDF)

S4 Table Allelic frequencies of candidate variants under the prioritization criteria identified with the ANNOVAR tool in AD family. (PDF)

S5 Table Results of evolutionary conservation predictors of candidate variants under the prioritization criteria identified with the ANNOVAR tool in AD family. (PDF)

S6 Table Pathogenicity predictors results of candidate variants under the prioritization criteria identified with the ANNOVAR tool in AD family. (PDF)

S7 Table Role of genes/proteins where candidate variants were identified in the family with Alzheimer’s disease. (PDF)

S8 Table Structure of genes and proteins where candidate variants were identified in the family with Alzheimer’s disease. (PDF)

S9 Table Energy and stereochemistry validation of structural models of SORL1 protein. (PDF)

S10 Table Energy and stereochemistry validation of structural models of MTHFD1L protein. (PDF)

S11 Table Primer information for sanger sequencing. (PDF)

S1 Appendix Clinical evaluation of index case (III:5). (PDF)

S2 Appendix Clinical evaluation of affected family member (III:10). (PDF)
