# Supplementary material for: Mutations in SORL1 and MTHFDL1 possibly contribute to the development of Alzheimer’s disease in a multigenerational Colombian Family
Source: PLoS One. 2022 Jul 29;17(7):e0269955. doi: 10.1371/journal.pone.0269955 (PMC9337667; doi:10.1371/journal.pone.0269955)
Supplement: S1 Fig — (PDF) [file pone.0269955.s001.pdf]

S1 Fig . Extended Pedigree of family with five generations.

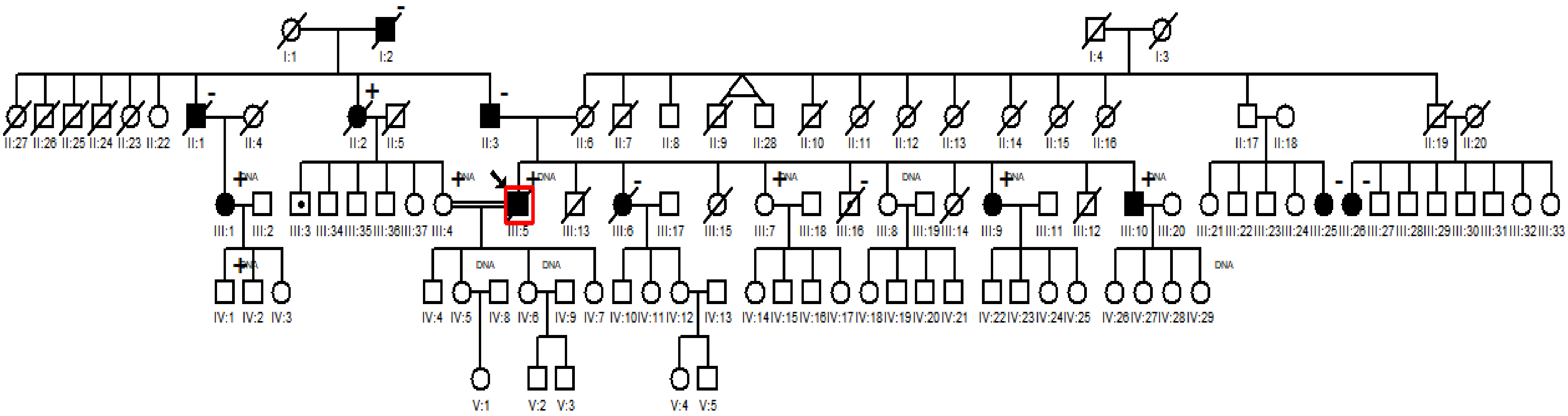

S1 Fig. Extended Pedigree of family with five generations. Black squares: Family members affected with AD. White squares: non-affected family members at time of death or last screening. **DNA**: Family members with DNA samples. **+** Family members with clinical evaluation.
