# Supplementary material for: Mutations in SORL1 and MTHFDL1 possibly contribute to the development of Alzheimer’s disease in a multigenerational Colombian Family
Source: PLoS One. 2022 Jul 29;17(7):e0269955. doi: 10.1371/journal.pone.0269955 (PMC9337667; doi:10.1371/journal.pone.0269955)
Supplement: S2 Fig — (PDF) [file pone.0269955.s002.pdf]

S2 Fig. Results obtained from the analysis of PSEN1 gene sequences in the family members affected with AD using Alivew software version 1.18.

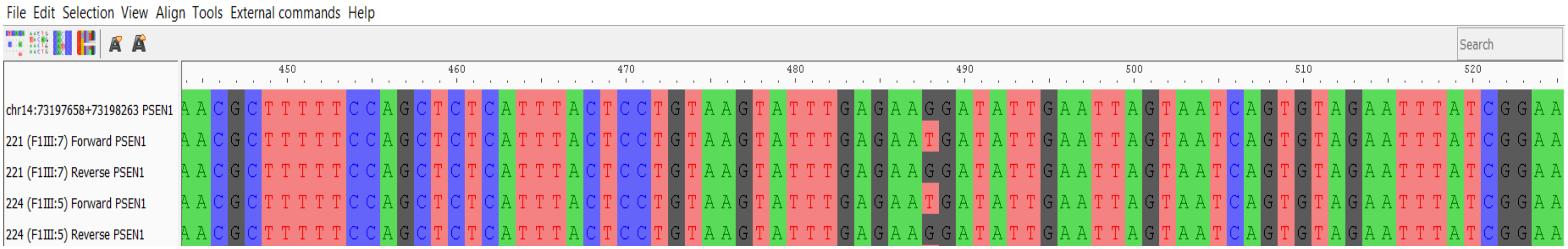

S2 Fig. Results obtained from the analysis of PSEN1 gene sequences in the family members affected with AD using Alivew software version 1.18. Affected family member (III:5) Heterozygous G/T. Healthy family member (III:7) Heterozygous G/T for the rs165932 polymorphism.
