# Supplementary material for: Mutations in SORL1 and MTHFDL1 possibly contribute to the development of Alzheimer’s disease in a multigenerational Colombian Family
Source: PLoS One. 2022 Jul 29;17(7):e0269955. doi: 10.1371/journal.pone.0269955 (PMC9337667; doi:10.1371/journal.pone.0269955)
Supplement: S4 Fig — (PDF) [file pone.0269955.s004.pdf]

S4 Fig. Classification of the genetic variants found in Family with AD using VEP tool.

Category	Count
Variants processed	71854
Variants filtered out	0
Novel / existing variants	68936 (95.9) / 2918 (4.1)
Overlapped genes	18025
Overlapped transcripts	79487
Overlapped regulatory features	12309

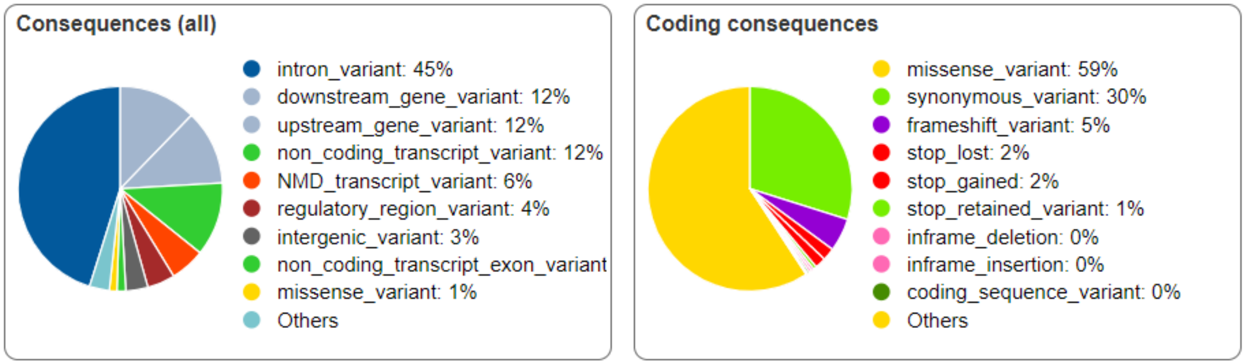

S4 Fig. Classification of the genetic variants found in Family with AD using VEP tool.
