# Supplementary material for: Mutations in SORL1 and MTHFDL1 possibly contribute to the development of Alzheimer’s disease in a multigenerational Colombian Family
Source: PLoS One. 2022 Jul 29;17(7):e0269955. doi: 10.1371/journal.pone.0269955 (PMC9337667; doi:10.1371/journal.pone.0269955)
Supplement: S5 Fig — (PDF) [file pone.0269955.s005.pdf]

S5 Fig. Protein-Protein Interaction Analysis using STRING tool.

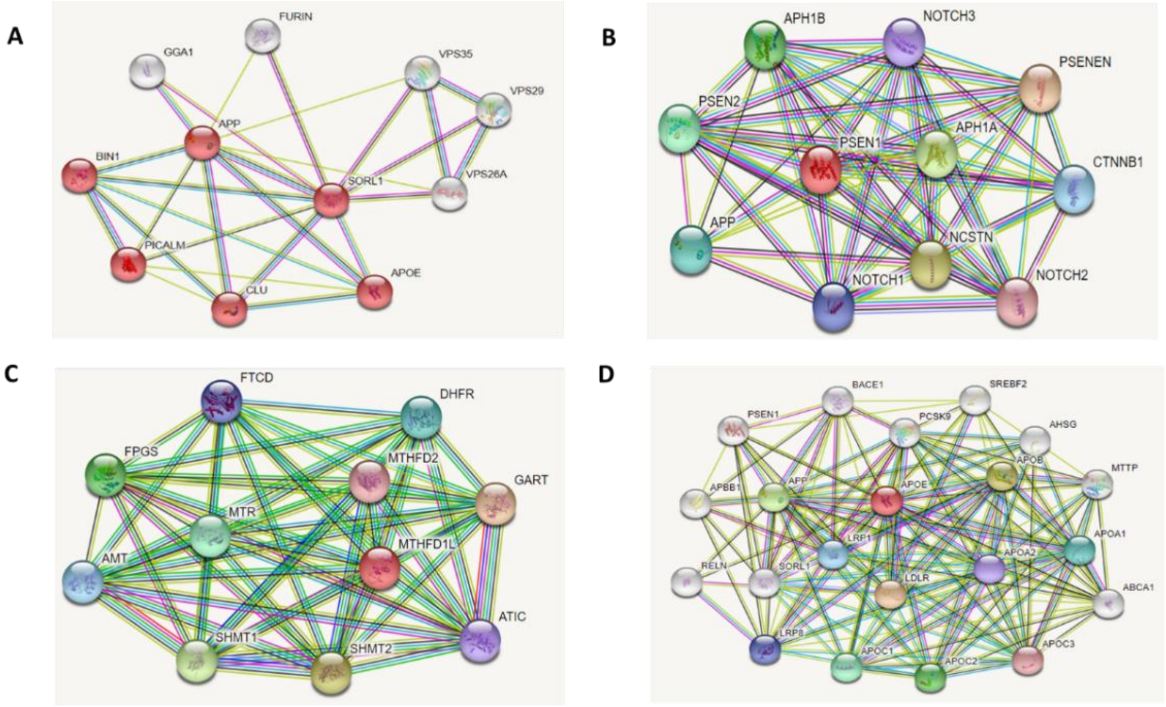

S5 Fig. Protein-Protein Interaction Analysis using STRING tool. A. SORL1 protein. B PSEN1 protein. C. MTHFD1L protein. D. APOE protein.
