# Supplementary material for: Mutations in SORL1 and MTHFDL1 possibly contribute to the development of Alzheimer’s disease in a multigenerational Colombian Family
Source: PLoS One. 2022 Jul 29;17(7):e0269955. doi: 10.1371/journal.pone.0269955 (PMC9337667; doi:10.1371/journal.pone.0269955)
Supplement: S6 Fig — (PDF) [file pone.0269955.s006.pdf]

S6 Fig. Structural Model of SORL1 protein.

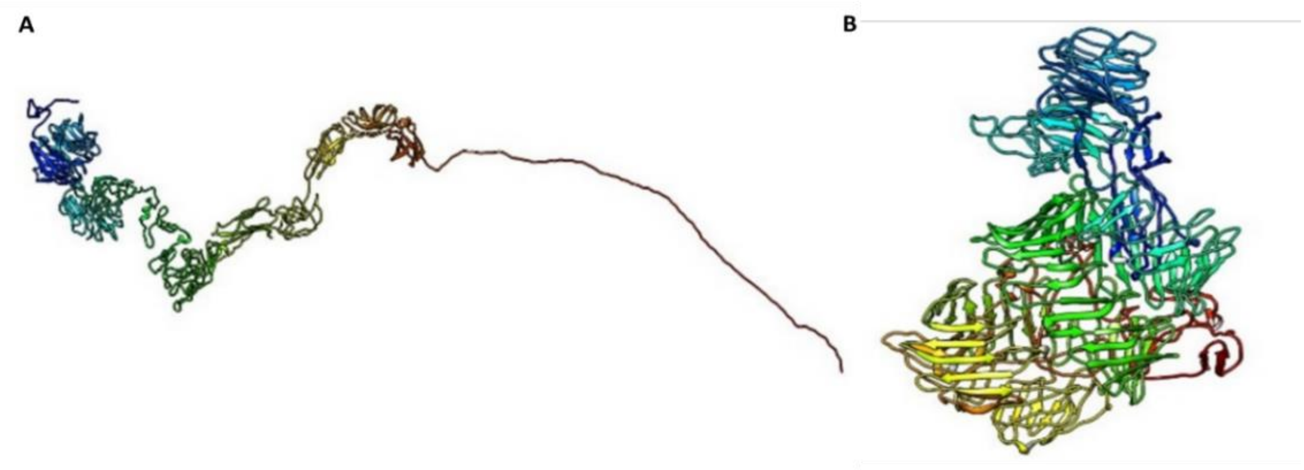

S6 Fig. Structural Model of SORL1 protein. A. SORL1 protein model builds with Phyre2 tool. B. SORL1 protein model builds with I-Tasser tool.
