# Supplementary material for: Mutations in SORL1 and MTHFDL1 possibly contribute to the development of Alzheimer’s disease in a multigenerational Colombian Family
Source: PLoS One. 2022 Jul 29;17(7):e0269955. doi: 10.1371/journal.pone.0269955 (PMC9337667; doi:10.1371/journal.pone.0269955)
Supplement: S7 Fig — (PDF) [file pone.0269955.s007.pdf]

S7 Fig. Ramachandran plot of SORL1 protein.

A

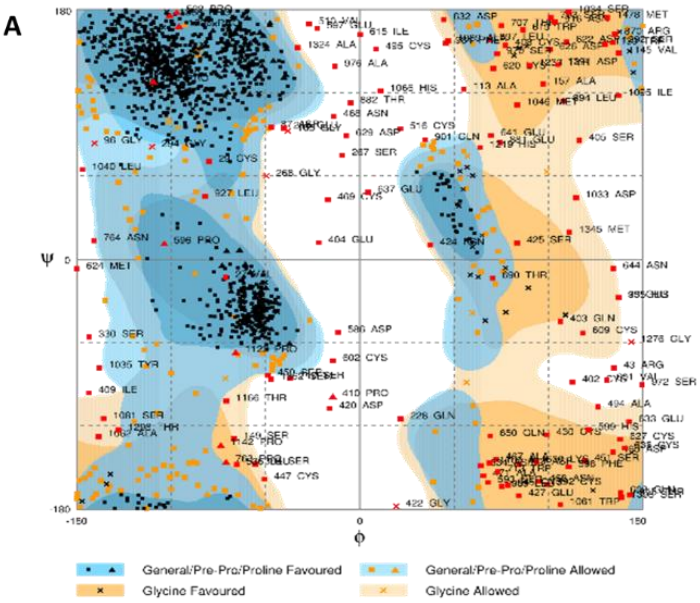

Number of residues in favoured region (~98.0% expected) : 1201 (80.7%)
Number of residues in allowed region (~2.0% expected) : 160 (10.7%)
Number of residues in outlier region : 128 (8.6%)

RAMPAGE by Paul de Bakker and Simon Lovell available at http://www-cryst.bioc.cam.ac.uk/rampage/
Please cite: S.C. Lovell, I.W. Davis, W.B. Arendall III, P.I.W. de Bakker, J.M. Word, M.G. Prisant, J.S. Richardson & D.C. Richardson (2002) Structure validation by Cα geometry: ϕ/ψ and Cβ deviation. *Proteins: Structure, Function & Genetics.* **50**: 437-450

B

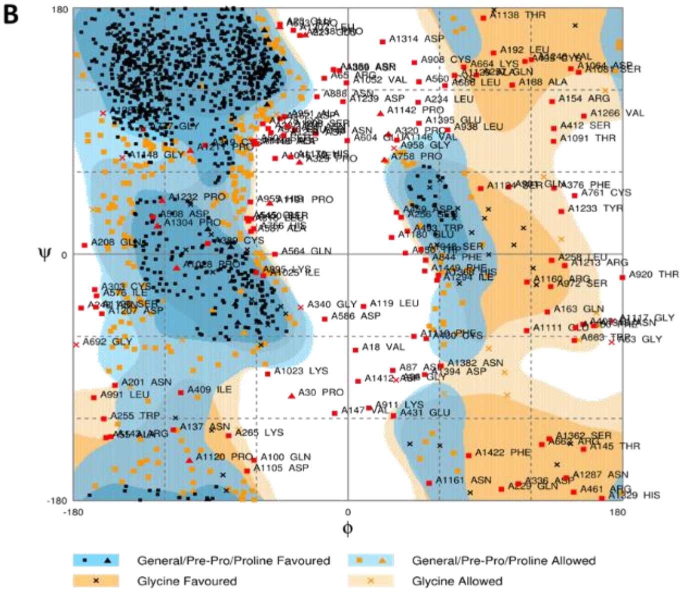

Number of residues in favoured region (~98.0% expected) : 1056 (70.9%)
Number of residues in allowed region (~2.0% expected) : 288 (19.3%)
Number of residues in outlier region : 145 (9.7%)

RAMPAGE by Paul de Bakker and Simon Lovell available at http://www-cryst.bioc.cam.ac.uk/rampage/
Please cite: S.C. Lovell, I.W. Davis, W.B. Arendall III, P.I.W. de Bakker, J.M. Word, M.G. Prisant, J.S. Richardson & D.C. Richardson (2002) Structure validation by Cα geometry: ϕ/ψ and Cβ deviation. *Proteins: Structure, Function & Genetics.* **50**: 437-450

S7 Fig. Ramachandran plot of SORL1 protein. A. SORL1 protein model builds with Phyre2 tool. Ramachandran plot shows that 80.7% of amino acids are in the favorable zone, 10.7% are in the allowed zone and 8.6% in the forbidden zone. B. SORL1 protein model builds with I-Tasser tool and refined with the Model Refiner tool. Ramachandran plot shows that 70.9% of amino acids are in the favorable zone, 19.3% are in the allowed zone and 9.7% in the forbidden zone.
