# Supplementary material for: Mutations in SORL1 and MTHFDL1 possibly contribute to the development of Alzheimer’s disease in a multigenerational Colombian Family
Source: PLoS One. 2022 Jul 29;17(7):e0269955. doi: 10.1371/journal.pone.0269955 (PMC9337667; doi:10.1371/journal.pone.0269955)
Supplement: S8 Fig — (PDF) [file pone.0269955.s008.pdf]

S8 Fig. Structural Model of MTHFD1L protein.

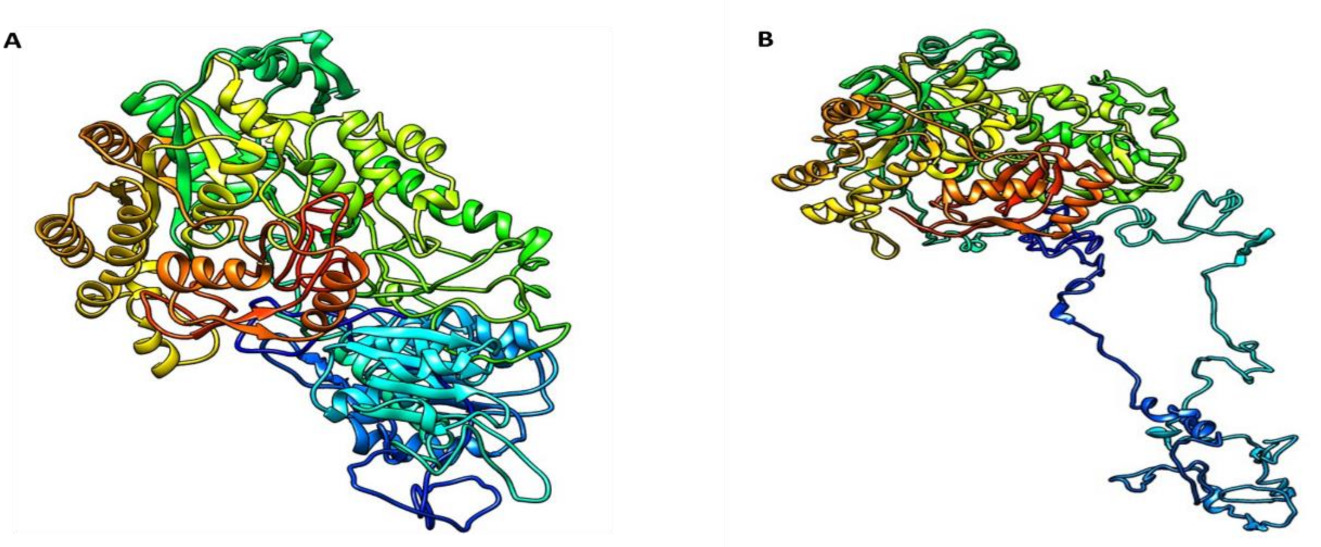

S8 Fig. Structural Model of MTHFD1L protein. A. MTHFD1L protein model builds with Phyre2 tool. B. MTHFD1L protein model builds with I-Tasser tool.
