# Supplementary material for: Mutations in SORL1 and MTHFDL1 possibly contribute to the development of Alzheimer’s disease in a multigenerational Colombian Family
Source: PLoS One. 2022 Jul 29;17(7):e0269955. doi: 10.1371/journal.pone.0269955 (PMC9337667; doi:10.1371/journal.pone.0269955)
Supplement: S9 Fig — (PDF) [file pone.0269955.s009.pdf]

S9 Fig. Ramachandran plot of MTHFD1L protein.

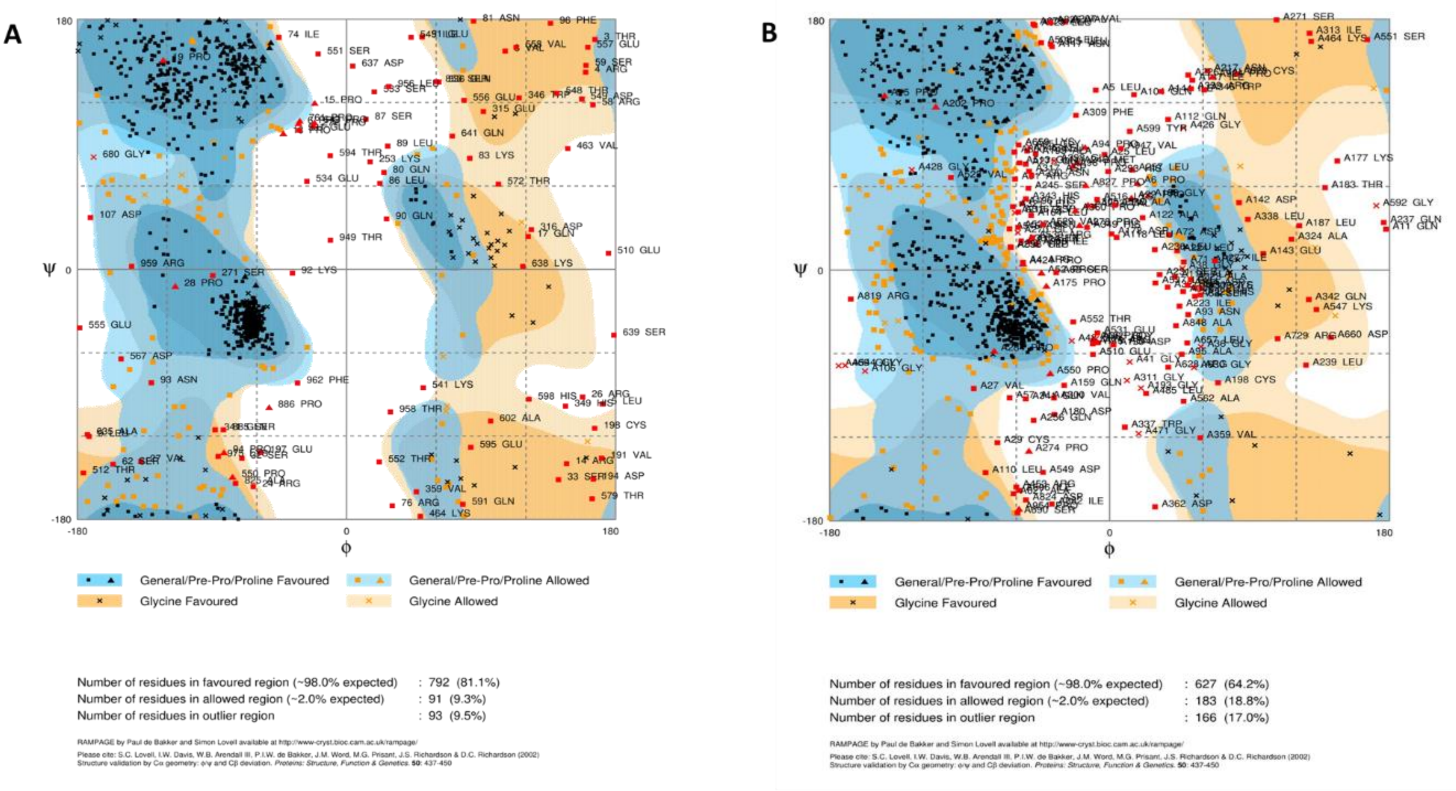

S9 Fig. Ramachandran plot of MTHFD1L protein. A. MTHFD1L protein model builds with Phyre2 tool. Ramachandran plot shows that 81.7% of amino acids are in the favorable zone, 9.3% are in the allowed zone and 9.5% in the forbidden zone. B. MTHFD1L protein model builds with I-Tasser tool and refined with the Model Refiner tool. Ramachandran plot shows that 64.2% of amino acids are in the favorable zone, 18.8% are in the allowed zone and 17.0% in the forbidden zone.
