# Supplementary material for: Mutations in SORL1 and MTHFDL1 possibly contribute to the development of Alzheimer’s disease in a multigenerational Colombian Family
Source: PLoS One. 2022 Jul 29;17(7):e0269955. doi: 10.1371/journal.pone.0269955 (PMC9337667; doi:10.1371/journal.pone.0269955)
Supplement: S1 Table — (PDF) [file pone.0269955.s010.pdf]

S1 Table. Evaluations carried out on members of Family with Familial Alzheimer's Disease.

ID	Status	Neurological evaluation	Neuropsychological evaluation	Imaging Evaluation	Histological Evaluation	DNA samples
I:2	Affected	No	No	No	No	No
I:1	Healthy	No	No	No	No	No
I:4	Healthy	No	No	No	No	No
I:3	Healthy	No	No	No	No	No
II:1	Affected	No	No	No	No	No
II:26	Healthy	No	No	No	No	No
II:24	Healthy	No	No	No	No	No
II:25	Healthy	No	No	No	No	No
II:23	Healthy	No	No	No	No	No
II:22	Healthy	No	No	No	No	No
II:27	Healthy	No	No	No	No	No
II:2	Affected	No	No	No	No	No
II:5	Healthy	No	No	No	No	No
II:3	Affected	No	No	No	No	No
II:6	Healthy	No	No	No	No	No
III:1	Affected	NO	NO	No	No	YES
III:2	Healthy	No	No	No	No	No
III:34	Healthy	No	No	No	No	No
III:35	Healthy	No	No	No	No	No
III:3	Uncertain	No	No	No	No	No
III:37	Healthy	No	No	No	No	No
III:36	Healthy	No	No	No	No	No
III:4	Healthy	**YES**	**YES**	No	No	**YES**
**III:5**	**Affected**	**YES**	**YES**	**Yes**	**Yes**	**YES**
III:12	Healthy	No	No	No	No	No
III:13	Healthy	No	No	No	No	No
III:14	Healthy	No	No	No	No	No
III:6	Affected	No	No	No	No	No
III:10	Affected	**YES**	**YES**	No	No	**YES**
III:15	Healthy	No	No	No	No	No
III:7	Healthy	**YES**	**YES**	No	No	**YES**
III:8	Healthy	No	No	No	No	**YES**
III:9	Affected	**YES**	**YES**	No	No	**YES**
III:11	Healthy	No	No	No	No	No
III:16	Uncertain	No	No	No	No	No
IV:2	Healthy	No	No	No	No	No
IV:3	Healthy	No	No	No	No	No
VI:1	Healthy	**YES**	**YES**	No	No	**YES**
IV:4	Healthy	No	No	No	No	No
IV:5	Healthy	No	No	No	No	**YES**
IV:6	Healthy	No	No	No	No	**YES**
IV:7	Healthy	No	No	No	No	**YES**
IV:29	Healthy	No	No	No	No	**YES**
IV:26	Healthy	No	No	No	No	No
IV:23	Healthy	No	No	No	No	No
IV:25	Healthy	No	No	No	No	No
IV:22	Healthy	No	No	No	No	No
IV:24	Healthy	No	No	No	No	No

S1 Table. Evaluations carried out on members of Family with Familial Alzheimer's Disease.
