# Supplementary material for: Mutations in SORL1 and MTHFDL1 possibly contribute to the development of Alzheimer’s disease in a multigenerational Colombian Family
Source: PLoS One. 2022 Jul 29;17(7):e0269955. doi: 10.1371/journal.pone.0269955 (PMC9337667; doi:10.1371/journal.pone.0269955)
Supplement: S2 Table — (PDF) [file pone.0269955.s011.pdf]

S2 Table. **Number of variants identified in the of variant calling and hard filtering process with the GATK program in a family with AD.**

ID	# Variant *(Variant calling)*				# Variant *(Hard filtering)*		# Var x Familia	
	TOTAL		SNPs	INDELs	SNPs	INDELs	SNPs	INDELs
224 (F1III:5)	71854	51521	47513	3995	40762	3124	57399	6193
221 (F1III:7)		53272	49086	4178	41171	3191		
222 (F1III:10)		54394	49654	4734	43569	3778		

S2 Table. **Number of variants identified in the of variant calling and hard filtering process with the GATK program in a family with AD**. **#Variants (variant calling):** Number of variants identified in the calling process variants (Variant calling - calling Haplotype). **#Variants (Hard filtering):** Number of variants identified after the filtering process. **#VarxFamily:** Number of variants identified in each family. **SNPs:** Single nucleotide polymorphisms**. INDELs:** Insertion/Deletion type variants.
