# Supplementary material for: Mutations in SORL1 and MTHFDL1 possibly contribute to the development of Alzheimer’s disease in a multigenerational Colombian Family
Source: PLoS One. 2022 Jul 29;17(7):e0269955. doi: 10.1371/journal.pone.0269955 (PMC9337667; doi:10.1371/journal.pone.0269955)
Supplement: S3 Table — (PDF) [file pone.0269955.s012.pdf]

S3 Table. Description of candidate variants under the prioritization criteria identified with the ANNOVAR tool in AD family.

Chr	Start	End	Ref	Alt	Region	Gene	Fun	AA Change	ClinVar Sig	ClinVar Disease	GWAS DIS	III:7	III:10	III:5
Chr11	121429346	121429346	C	T	exonic	SORL1	nonsyn	exon20 C2710T R904W	.	.	.	0/0	0/0	0/1
Chr17	44073870	44073870	G	C	exonic	MAPT	nonsyn	exon11 G1667C R556P	.	.	.	0/0	0/1	0/0
Chr10	50854563	50854563	G	A	exonic	CHAT	nonsyn	exon8 G770A R257Q	.	.	.	0/0	0/0	0/1
Chr19	1058841	1058841	C	-	exonic	ABCA7	Frameshift deletion	exon39 5302delC L1768fs	.	.	.	0/1	0/0	0/1
Chr19	1043103	1043103	G	A	exonic	ABCA7	nonsyn	exon8 G643A G215S	.	.	.	0/0	0/0	0/1
Chr19	1050996	1050996	G	A	exonic	ABCA7	nonsyn	exon19 G2629A A877T	.	.	.	0/1	0/0	0/1
Chr6	160961137	160961137	T	C	exonic	LPA	nonsyn	exon37 A5673G I1891M	.	.	Coronary heart disease	0/0	0/1	0/1
Chr6	151270231	151270231	G	A	exonic	MTHFD1L	non syn	exon16 G1691A R564H	.	.	.	0/0	0/1	0/0
Chr19	45411941	45411941	T	C	exonic	APOE	non syn	exon4 T388C C130R	Pathogenic Pathogenic Pathogenic Pathogenic Other other	Familial type 3 hyperlipoproteinemia Alzheimer disease 2 Familial type 3 hyperlipoproteinemia APOE4(-)-FREIBURG APOE4 VARIANT not provided	Alzheimer's disease biomarkers	0/1	1/1	0/1

S3 Table. Description of candidate variants under the prioritization criteria identified with the ANNOVAR tool in AD family. Chr: Chromosome. **Start:** Variant start position. **End:** Variant end position. **Ref:** Reference allele. **Alt:** Alternate allele. **Region:** Gene region (exonic, intronic, intergenic, UTR). **Gene:** Gene name. **Fun:** Function (synonymous, non-synonymous Frameshift). **AA Change:** Amino acid change. **ClinVarSig:** Clinical significance in ClinVar database (Benign, Likely benign, Uncertain, Likely pathogenic, Pathogenic, Other) **ClinVarDisease:** Medical conditions reported for that variant. **GWASDIS:** Associated disease in GWAS Catalog database. **III:7:** non-affected family member. **III:10:** affected family member. **III:5:** affected family member. Genotype: 0=Reference allele, 1=Alternate allele.
