# Supplementary material for: Mutations in SORL1 and MTHFDL1 possibly contribute to the development of Alzheimer’s disease in a multigenerational Colombian Family
Source: PLoS One. 2022 Jul 29;17(7):e0269955. doi: 10.1371/journal.pone.0269955 (PMC9337667; doi:10.1371/journal.pone.0269955)
Supplement: S4 Table — (PDF) [file pone.0269955.s013.pdf]

S4 Table. Allelic frequencies of candidate variants under the prioritization criteria identified with the ANNOVAR tool in AD family.

Chr	Ref	Alt	Gene	dbSNP	1000G ALL	1000G AMR	1000G CLM	ExAC Freq	ExAC AMR	ESP 6500si ALL	gnomAD exome ALL	gnomAD exome AMR	gnomAD genome ALL	gnomAD genome AMR	III:7	III:10	III:5
Chr11	C	T	SORL1	rs148966249	0.0002	0.0014	**0.0053**	4.119e-05	8.642e-05	0.0001	1.219e-05	2.978e-05	.	.	0/0	0/0	0/1
Chr17	G	C	MAPT	.	.	.	.	.	.	.	.	.	.	.	0/0	0/1	0/0
Chr10	G	A	CHAT	rs201616704	0.0002	0.0014	0.0053	0.0003	8.646e-05	0.0001	0.0003	8.934e-05	3.233e-05	0	0/0	0/0	0/1
Chr19	C	-	ABCA7	.	.	.	.	.	.	.	4.532e-06	3.34e-05	.	.	0/1	0/0	0/1
Chr19	G	A	ABCA7	rs72973581	0.02	0.02	0.0160	0.0432	0.0175	0.046	0.0424	172	0.0465	0.0239	0/0	0/0	0/1
Chr19	G	A	ABCA7	rs74176364	0.023	0.13	0.1011	0.0169	0.0970	0.0052	0.0207	970	0.0103	0.0819	0/1	0/0	0/1
Chr6	T	C	LPA	rs3798220	0.051	0.22	0.1915	0.0448	0.2901	0.016	.	0.0076	0.0039	0.0080	0/0	0/1	0/1
Chr6	G	A	MTHFD1L	rs61748674	0.0062	0.0086	**0.0160**	0.0127	0.0060	0.013	.	0.0032	0.0105	0.0033	0/0	0/1	0/0
Chr19	T	C	APOE	rs429358	0.15	0.1	0.1543	0.1843	0.2145	0.14	.	2223	1006	0.2110	0/1	1/1	0/1

S4 Table. Allelic frequencies of candidate variants under the prioritization criteria identified with the ANNOVAR tool in AD family. **Chr:** Chromosome. **Ref:** Reference allele. **Alt:** Alternate allele. **Gene:** Gene name. **dbSNP:** Variant identifier in dbSNP database. **1000GALL:** Allele frequency in 1000 genomes data base (all populations). **1000GAMR:** Allele frequency in 1000 genomes data base (American population). **1000GCLM:** Allele frequency in 1000 genomes data base (Colombian population). **ExACFreq:** Allele frequency in ExAC 65000 data base (all populations). **ExACAMR:** Allele frequency in ExAC 65000 data base (American population). **ESP6500siALL:** Allele frequency in NCBI-ESP 6500 database (all populations). **gnomADexomeALL:** Allele frequency in gnomAD database, exome data (all populations). **gnomADexomeAMR:** Allele frequency in gnomAD database, exome data (American population). **gnomADgenomeALL:** Allele frequency in gnomAD database, genome data (all populations). **gnomADgenomeAMR:** Allele frequency in gnomAD database, genome data (American population). **III:7:** non-affected family member. **III:10:** affected family member. **III:5:** affected family member. Genotype: 0=Reference allele, 1=Alternate allele.
