# Supplementary material for: Mutations in SORL1 and MTHFDL1 possibly contribute to the development of Alzheimer’s disease in a multigenerational Colombian Family
Source: PLoS One. 2022 Jul 29;17(7):e0269955. doi: 10.1371/journal.pone.0269955 (PMC9337667; doi:10.1371/journal.pone.0269955)
Supplement: S5 Table — (PDF) [file pone.0269955.s014.pdf]

S5 Table. Results of evolutionary conservation predictors of candidate variants under the prioritization criteria identified with the ANNOVAR tool in AD family.

Chr	Ref	Alt	Gene	dbSNP	Geno Canyon	fitCons	GERP++RS	phyloP vertebrate	phyloP mammalian	phastCons vertebrate	phastCons mammalian	SiPhy	III:7	III:10	III:5
chr11	C	T	SORL1	rs148966249	1.000	0.722	3.65	1.608	0.059	1.000	0.981	10.390	0/0	0/0	0/1
chr17	G	C	MAPT	.	0.996	0.581	5.62	0.969	0.958	0.990	0.822	18.220	0/0	0/1	0/0
chr10	G	A	CHAT	rs201616704	1.0	0.497	5.16	9.998	0.953	1.000	1.000	18.645	0/0	0/0	0/1
chr19	C	-	ABCA7	.	.	.	.	.	.	.	.	.	0/1	0/0	0/1
chr19	G	A	ABCA7	rs72973581	1.000	0.651	-5.86	0.414	0.007	0.000	0.001	5.684	0/0	0/0	0/1
chr19	G	A	ABCA7	rs74176364	1.000	0.696	2.59	1.978	0.076	0.988	0.063	10.280	0/1	0/0	0/1
chr6	T	C	LPA	rs3798220	0.003	0.487	2.99	0.210	0.724	0.000	0.151	9.750	0/0	0/1	0/1
chr6	G	A	MTHFD1L	rs61748674	1.000	0.719	5.9	9.569	1.029	1.000	0.791	20.260	0/0	0/1	0/0
chr19	T	C	APOE	rs429358	0.194	0.635	3.02	0.840	-0.026	0.892	0.182	3.038	0/1	1/1	0/1

S5 Table. Results of evolutionary conservation predictors of candidate variants under the prioritization criteria identified with the ANNOVAR tool in AD family. Chr: Chromosome. **Ref:** Reference allele. **Alt:** Alternate allele. **Gene:** Gene name. **dbSNP:** Variant identifier in dbSNP database. Evolutionary conservation predictors scores. **GenoCanyon:** Conservation scores with GenoCanyons tool (Conserved region=scores~1). **fitCons:** Conservation scores with fitCons tool: (Conserved region= ~1). **GERP++RS:** Conservation scores with GERP++RS tool (Conservation region=scores>4.4) **phyloPvertebrate:** Conservation scores with phyloP100 tool for vertebrates (Conservation region=scores>1.6). **phyloPmammalian:** Conservation scores with phyloP100 tool for mammalian (Conservation region=scores>1.6). **phastConsvertebrate:** Conservation scores with phastCons tool for vertebrates (Conservation region= scores~1). **phastConsmammalian:** Conservation scores with phastCons tool for vertebrates (Conservation region= scores~1) **SiPhy:** Conservation scores with SiPhy tool (Conservation region=scores >12.17). III:7: non-affected family member. III:10: affected family member. III:5: affected family member. Genotype: 0=Reference allele, 1=Alternate allele.
