# Supplementary material for: Mutations in SORL1 and MTHFDL1 possibly contribute to the development of Alzheimer’s disease in a multigenerational Colombian Family
Source: PLoS One. 2022 Jul 29;17(7):e0269955. doi: 10.1371/journal.pone.0269955 (PMC9337667; doi:10.1371/journal.pone.0269955)
Supplement: S6 Table — (PDF) [file pone.0269955.s015.pdf]

S6 Table. Pathogenicity predictors results of candidate variants under the prioritization criteria identified with the ANNOVAR tool in AD family.

Chr	Gene	dbSNP	SIFT	Poly phen2 HDIV	Poly phen2 HVAR	LRT	Mutation Taster	Mutation Assessor	FATHMM	PROVEAN	VEST3	Meta SVM	Meta LR	M-CAP	CADD	DANN score	Fathmm MKL	III:7	III:10	III:5
**chr11**	**SORL1**	**rs148966249**	**D**	**D**	**D**	**N**	**D**	**M**	**D**	**D**	**0.911**	**D**	**D**	**D**	**33**	**0.999**	**D**	**0/0**	**0/0**	**0/1**
**chr17**	**MAPT**	**.**	**D**	**D**	**D**	**N**	**D**	**L**	**T**	**D**	**0.743**	**T**	**T**	**D**	**24.5**	**0.997**	**D**	**0/0**	**0/1**	**0/0**
**chr10**	**CHAT**	**rs201616704**	**D**	**P**	**B**	**D**	**D**	**M**	**D**	**N**	**0.56**	**D**	**D**	**D**	**25.4**	**0.999**	**D**	**0/0**	**0/0**	**0/1**
chr19	ABCA7	.	.	.	.	.	.	.	.	.	.	.	.	.	.	.	.	0/1	0/0	0/1
chr19	ABCA7	rs72973581	T	B	B	.	N	L	D	N	0.04	T	T	.	0.004	0.480	N	0/0	0/0	0/1
**chr19**	**ABCA7**	**rs74176364**	**D**	**B**	**B**	**.**	**N**	**L**	**D**	**N**	**0.111**	**T**	**T**	**.**	**19.47**	**0.954**	**D**	**0/1**	**0/0**	**0/1**
chr6	LPA	rs3798220	T	D	D	.	P	L	D	N	0.09	T	T	.	16.65	0.970	N	0/0	0/1	0/1
**chr6**	**MTHFD1L**	**rs61748674**	**D**	**D**	**D**	**D**	**D**	**H**	**T**	**D**	**0.31**	**T**	**T**	**.**	**31**	**0.999**	**D**	**0/0**	**0/1**	**0/0**
chr19	APOE	rs429358	T	B	B	N	P	N	T	N	0.087	T	T	.	0.007	0.217	N	0/1	1/1	0/1

S6 Table. Pathogenicity predictors results of candidate variants under the prioritization criteria identified with the ANNOVAR tool in AD family. Chr: Chromosome. **Gene:** Gene name. **dbSNP:** Variant identifier in dbSNP database. Pathogenicity predictors scores. **SIFT:** Pathogenicity prediction with SIFT tool: D=Deleterious, T=Tolerated). **Polyphen2HDIV:** Pathogenicity prediction with PolyPhem2 tool for Mendelian disease variants (D=Damaging, P=Possibly Damaging, B=Benign, U=Unknown). **Polyphen2HVAR:** Pathogenicity prediction with PolyPhem2 tool for all human disease-causing mutations (D=Damaging, P=Possibly Damaging, B=Benign, U= Unknown). **LRT:** Pathogenicity prediction with LTR tool (D=Deleterious, N=No Deleterious). **MutationTaster:** Pathogenicity prediction with Mutation Tester tool (A=Disease causing automatic, D=Disease causing, N=Polymorphism, P= Polymorphism automatic). **MutationAssessor:** Pathogenicity prediction with Mutation Assessor tool (N= Neutral effect, L=Low effect, M=Medium effect, H=High effect). **FATHMM:** Pathogenicity prediction with FATHMMM tool (D=Deleterious, T=Tolerated). **PROVEAN:** Pathogenicity prediction with PROVEAN tool (D=Deleterious, N=No Deleterious). **VEST3:** Pathogenicity SCORES with VEST tool (Deleterious=scores>0.63). **MetaSVM:** Pathogenicity prediction with MetaSVM tool (D=Damaging, T=Tolerated). **MetaLR:** Pathogenicity prediction with MetaLR tool (D=Damaging, T=Tolerated). **M-CAP:** Pathogenicity prediction with M-CAP tool (D=Damaging, B=Benign). **CADD:** Pathogenicity scores with CADD tool (Deleterious=scores>14). **DANN:** Pathogenicity prediction with DANN tool (Pathogenic= scores~1). **FathmmMKL:** Pathogenicity prediction with FathmmMKL tool (D=Deleterious, T=Tolerated). **III:7:** non-affected family member. **III:10:** affected family member. **III:5:** affected family member. Genotype: 0=Reference allele, 1=Alternate allele.
