# Supplementary material for: Mutations in SORL1 and MTHFDL1 possibly contribute to the development of Alzheimer’s disease in a multigenerational Colombian Family
Source: PLoS One. 2022 Jul 29;17(7):e0269955. doi: 10.1371/journal.pone.0269955 (PMC9337667; doi:10.1371/journal.pone.0269955)
Supplement: S7 Table — (PDF) [file pone.0269955.s016.pdf]

S7 Table. Role of genes/proteins where candidate variants were identified in the family with Alzheimer's disease.

Gene name and ID	Protein name and ID	Gene information from (NCBI) https://www.ncbi.nlm.nih.gov	Gene information from (GeneCards) https://www.genecards.org/	Protein information from (UniProt) https://www.uniprot.org/
**SORL1** ID: 6653	Sortilin-related receptor) ID: Q92673	This gene encodes a protein that belongs to at least two families: the receptor family that contains the vacuolar protein domain (VPS10) and the low-density lipoprotein receptor (LDLR) family. It also contains repeats of type III fibronectin and a repeat of epidermal growth factor. The preprotein is processed proteolytically to generate the mature receptor, which probably plays a role in endocytosis and classification. Mutations in this gene may be associated with Alzheimer's disease.	SORL1-associated diseases include autosomal dominant early-onset Alzheimer's disease and Alzheimer's disease. Among its related pathways are the PKN1 activation pathway that stimulates the transcription of the genes regulated by AR (androgen receptor) KLK2 and KLK3 and the metabolism of proteins. Gene Ontology (GO) annotations related to this gene include transmembrane signaling receptor activity and binding to low-density lipoprotein particles.	Multifunctional endocytic receptor involved in the uptake of lipoproteins and proteases. It binds to LDL and facilitates its transport to cells by endocytosis. Participate in APP traffic to and from the Golgi apparatus. Therefore, it probably acts as a sorting receptor that protects APP from late endosome trafficking and beta-amyloid peptide processing, thereby reducing the burden of amyloidogenic peptide formation. It also participates in the regulation of the migration of smooth muscle cells.
**MAPT** ID: 4137	Microtubule-associated protein tau) ID: P10636	This gene encodes the microtubule-associated protein tau (MAPT) whose transcription undergoes a complex and regulated alternative splicing, which gives rise to several species of mRNA. MAPT transcripts are differentially expressed in the nervous system, depending on the stage of neuronal maturation and the type of neuron. MAPT gene mutations have been associated with various neurodegenerative disorders such as Alzheimer's disease, Pick's disease, frontotemporal dementia, cortico-basal degeneration, and progressive supranuclear palsy.	MAPT-associated diseases include frontotemporal dementia and Pick's disease. Among its related pathways are the Kit receptor signaling pathway and the IL-2 signaling pathway. Gene Ontology (GO) annotations related to this gene include protein kinase binding and microtubule binding.	It promotes the assembly and stability of the microtubules. Involved in the establishment and maintenance of neural polarity. The C terminus binds to axonal microtubules, while the N terminus binds to components of the neural plasma membrane, suggesting that tau functions as a linker protein between the two. Axonal polarity is predetermined by the location of TAU / MAPT in the domain of the cell body defined by the centrosome. Short isoforms allow the plasticity of the cytoskeleton, while longer isoforms may play a preferential role in its stabilization.
**CHAT** ID: 1103	Choline O-acetyltransferase ID: P28329	This gene encodes an enzyme that catalyzes the biosynthesis of the neurotransmitter acetylcholine. This gene product is a characteristic trait of cholinergic neurons, and changes in these neurons may explain some of the symptoms of Alzheimer's disease. Polymorphisms in this gene have been associated with Alzheimer's disease, mild cognitive decline, and congenital myasthenic syndrome associated with episodic apnea.	Diseases associated with CHAT include myasthenic, congenital, presynaptic syndrome 6, and central sleep apnea. Among its related pathways are the metabolism and biosynthesis of glycerophospholipids. Gene Ontology (GO) annotations related to this gene include transferase activity, acyl group transfer, and choline O-acetyltransferase activity.	It catalyzes the reversible synthesis of acetylcholine (ACh) from acetyl CoA and choline at cholinergic synapses.
**ABCA7** ID: 10347	ATP binding cassette subfamily A member 7 ID: Q8IZY2	The protein encoded by this gene is a member of the ATP-binding transporter (ABC) superfamily, which transport various molecules across extracellular and intracellular membranes. The ABC genes are divided into seven distinct subfamilies (ABC1, MDR / TAP, MRP, ALD, OABP, GCN20, White). This protein is a member of the ABC1 subfamily, which constitute the only subfamily found exclusively in multicellular eukaryotes. This transporter has been detected predominantly in myelo-lymphatic tissues with greater expression in peripheral leukocytes, thymus, spleen, and bone marrow.	ABCA7-associated diseases include Alzheimer's disease 9 and conjunctival folliculosis. Among its related routes are the transport of Cholesterol and Sphingolipids. Recycling of the plasma membrane in the lung, CDK-mediated phosphorylation and Cdc6 removal. Gene Ontology (GO) annotations related to this gene include transporter activity and ATPase activity, associated with the transmembrane movement of substances.	It plays a role in lipid homeostasis and macrophage-mediated phagocytosis. It binds to APOA1 and facilitates the outflow of phospholipids and cholesterol mediated by apolipoproteins. It can regulate the homeostasis of cellular ceramide during keratinocyte differentiation. Participates in the organization of lipid rafts. It plays a role in macrophage apoptotic cell phagocytosis. Participates in the sweep of the amyloid beta peptide by microglial cells and macrophages. It limits the production of beta amyloid by playing a role in the regulation of endocytosis and / or processing of APP.
**LPA** ID: 4018	Lipoprotein A ID: P08519	The protein encoded by this gene is a serine proteinase that inhibits tissue type I plasminogen activator. The encoded protein constitutes a substantial portion of lipoprotein (a) that is proteolytically cleaved, resulting in fragments that bind to atherosclerotic lesions and promote thrombogenesis. Elevated plasma levels of this protein are related to atherosclerosis. Depending on the individual, the encoded protein contains 2-43 copies of kringle-like domains.	The diseases associated with LPA include pancreatic sarcoma and peripheral vascular disease. Among its related pathways are lipoprotein metabolism. Gene Ontology (GO) annotations related to this gene include serine-like endopeptidase activity and endopeptidase inhibitory activity.	Apo (a) is the main constituent of lipoprotein (a). It has serine proteinase activity and is capable of self-protection. It is cleaved proteolytically forming the so-called mini-Lp (a). Apo (a) fragments accumulate in atherosclerotic lesions, where they can promote thrombogenesis. Inhibits tissue-type plasminogen activator 1. Homology with kringle domains IV and V of plasminogen underlies the atherogenicity of the protein since the fragments compete with plasminogen for binding of fibrin.
**MTHFD1L** ID: 25902	Methylenetetrahydrofolate dehydrogenase (NADP+ dependent) 1 like ID: Q6UB35	The protein encoded by this gene is involved in the synthesis of tetrahydrofolate (THF) in the mitochondria. THF is important in de novo synthesis of purines and thymidylate and in the regeneration of methionine from homocysteine. Several transcription variants have been found that encode different isoforms for this gene.	Diseases associated with MTHFD1L include colon adenocarcinoma and neural tube defects. Among its related pathways are folate metabolism. Gene Ontology (GO) annotations related to this gene include protein homodimerization activity and methenyltetrahydrofolate cyclohydrolase activity.	It can provide the missing metabolic reaction necessary to link mitochondria and cytoplasm in the mammalian model of folate metabolism in transformed and embryonic cells, thus complementing the enzymatic activities of MTHFD2. It can participate in the progression of colorectal cancer by conferring a growth advantage. It could be a new molecular target for cancer therapy.
**APOE** ID: 348	Apolipoprotein E ID: P02649	The protein encoded by this gene is the main apoprotein of the chylomicron. It binds to	Diseases associated with APOE include lipoprotein glomerulopathy and type III hyperlipoproteinemia.	It is a protein associated with lipid particles, which works primarily in lipoprotein mediated

		peripheral and liver-specific cellular receptors and is essential for the normal catabolism of triglyceride-rich lipoprotein components. This gene is located on chromosome 19 along with the genes related to apolipoprotein C1 and C2. Mutations in this gene give rise to familial dysbetalipoproteinemia, or type III hyperlipoproteinemia (HLP III), in which the increase in plasma cholesterol and triglycerides is the consequence of poor scanning of the chylomicron and VLDL residues.	Among its related pathways are metabolism and the apoptosis-related network due to the alteration of Notch3 in ovarian cancer. Gene Ontology (GO) annotations related to this gene include protein homodimerization activity and binding to signaling receptors.	lipid transport between organs through plasma and interstitial fluids. Central component of plasma lipoproteins involved in its production, conversion, and purification. It is associated with chylomicrons, chylomicron remnants, VLDL, and IDL, but shows preferential HDL binding. It also binds to a wide range of cellular LDL / LDLR receptors, LRP1, LRP2 and LRP8, and VLDLR that mediates cellular uptake of APOE-containing lipoprotein particles.

S7 Table. Role of genes/proteins where candidate variants were identified in the family with Alzheimer's disease.
