# Supplementary material for: Mutations in SORL1 and MTHFDL1 possibly contribute to the development of Alzheimer’s disease in a multigenerational Colombian Family
Source: PLoS One. 2022 Jul 29;17(7):e0269955. doi: 10.1371/journal.pone.0269955 (PMC9337667; doi:10.1371/journal.pone.0269955)
Supplement: S8 Table — (PDF) [file pone.0269955.s017.pdf]

S8 Table. Structure of genes and proteins where candidate variants were identified in the family with Alzheimer's disease.

Gene name and ID	Protein name and ID	Gene and Protein Structure
**SORL1**<br>ID: 6653	Sortilin-related receptor)<br>ID: Q92673	The SORL1 gene is located in the 11q24.1 region, is 188511 base pairs in size and is made up of 53 exons. This gene codes for a transmembrane type I protein, SorL1 of 250 kDa and 2,214 amino acids and is a member of the family of low-density lipoprotein receptors. Its structure consists of several domains with different functions: an N-terminal domain VPS10 (vacuolar protein classification domain) important for the classification and transport of endosomal proteins and can also interact with different neuropeptides and participate in APP processing, 5 domains LDL-receptor class B (low-density lipoprotein receptor YWTD domain) that plays a central role in cholesterol metabolism, an epidermal growth factor-like domain that plays a vital role in immune response as well as extraction of dead cells in the body, 11 class A receptor-LDL domains, identified as the lipoprotein binding site and 6 type III fibronectin domains, involved in processes of cell adhesion, cell morphology, thrombosis, cell migration and embryonic differentiation. The C2710T variant is found in exon 20 of the gene and the R904W change is located in the extracellular region of the protein, specifically in the third LDL-receptor class B domain that extends from amino acid 888 to 932, important in metabolism cholesterol and APP binding site.
	Structure of the SORL1 gene taken from the Ensembl platform.	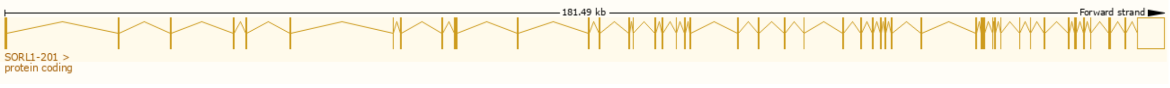
	Structure of the SORL1 protein taken from the SMART platform.	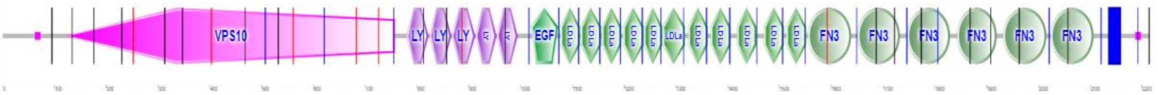
**MAPT**<br>ID: 4137	Microtubule-associated protein tau)<br>ID: P10636	The MAPT gene is located in the 17q21.31 region, has a size of 140924 base pairs and is made up of 15 exons. This gene codes for the Tau microtubule-associated protein of 78 kDa and 758 amino acids. Its structure is characterized by the presence of four tubulin binding repeats at the C-terminus, Tau / MAP 1 (561-591), Tau / MAP 2 (592-622), Tau / MAP 3 (623-653) and Tau / MAP 4 (654 - 685), important for tubulin binding. The G1667C variant is found in exon 11 of the gene and the R556P change is located at the C-terminus of the protein, near the first Tau / MAP 1 repeat that extends from amino acid 561 to 591, important for its function of binding to tubulin.
	Structure of the MAPT gene taken from the Ensembl platform.	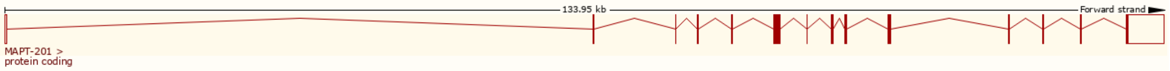
	Structure of the Tau protein taken from the Prosite platform.	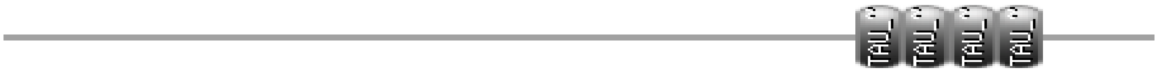
**CHAT**<br>ID: 1103	Choline O-acetyltransferase<br>ID: P28329	The CHAT gene is located in the 10q11.23 region, is 63010 base pairs in size and is made up of 15 exons. This gene codes for the 83 kDa 748 amino acid ChAT enzyme. Its structure consists of a colin/carnitine acetyltransferase domain, which participates in the transfer of an acyl group from one compound (donor) to another (acceptor). The G1124A variant is found in exon 8 of the gene and the R375Q change is located within the colin / carnitine acetyltransferase domain that extends from amino acid 131 to 719 and about 145 amino acids from the Coenzyme A binding site that covers 13 amino acids from the 520 to 532 of the protein.
	Structure of the CHAT gene taken from the Ensembl platform.	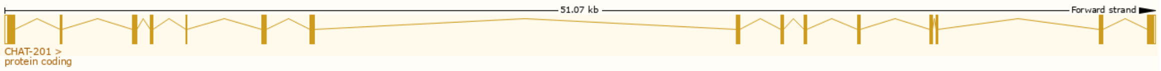
	Structure of the CHAT protein taken from the Pfam platform.	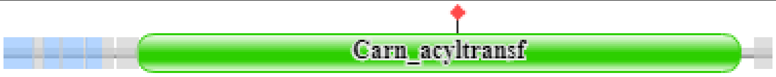
**ABCA7**<br>ID: 10347	ATP binding cassette subfamily A member 7<br>ID: Q8IZY2	The ABCA7 gene is located in region 19p13.3, is 25,470 base pairs in size and is made up of 47 exons Fig #. This gene codes for the ABCA7 protein of 234 kDa and 2,146 amino acids. Its structure consists of two highly conserved ATP binding domains (ATPase domain), the first (ABC transporter 1) located from amino acid 807 to 1038 and the second (ABC transporter 2) located from amino acid 1793 - 2025, which use energy from ATP hydrolysis for export or import of a wide variety of substrates ranging from small ions to macromolecules. The G2629A variant is found in exon 19 of the gene and the A877T change is located in the first ATP binding domain important for ATP hydrolysis.
	Structure of the ABCA7 gene taken from the Ensembl platform.	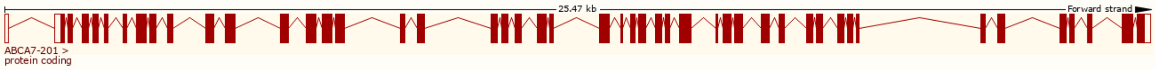
	Structure of the ABCA7 protein taken from the Pfam platform.	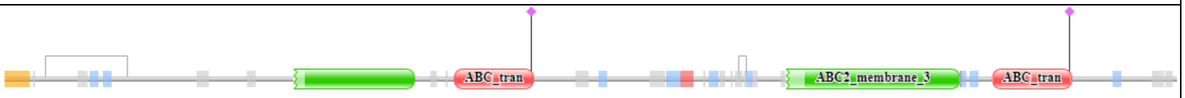
LPA<br>ID: 4018	Lipoprotein A<br>ID: P08519	The LPA gene is located in the 6q25.3-q26 region, is 134893 base pairs in size and is made up of 40 exons. This gene codes for Apolipoprotein A of 501 kDa and 4,548 amino acids. This protein is made up of 38 Kringle-like domains, which play a role as binding mediators (for example, in membranes, proteins or

		phospholipids) and in the regulation of proteolytic activity and a Trypsin domain at the C-terminus containing the active site with serine-like endopeptidase activity. The A5673G variant is located in exon 37 of the gene and the I1891M change is located in one of the Kringle-like domains.
	Structure of the LPA gene taken from the Ensembl platform.	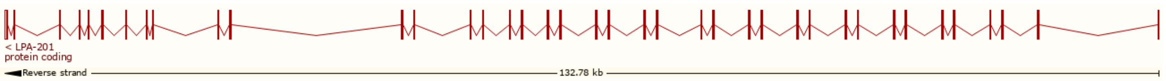
	Structure of the LPA protein taken from the Prosite platform.	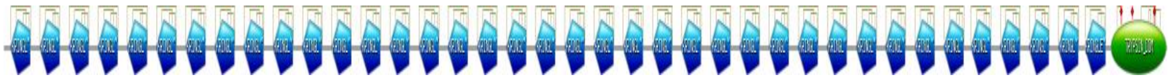
MTHFD1L<br>ID: 25902	Methylenetetrahydrofolate dehydrogenase (NADP+ dependent) 1 like<br>ID: Q6UB35	The MTHFD1L gene is located in the 6q25.1 region, is 236209 base pairs in size and is made up of 42 exons. This gene codes for the enzyme C1-tetrahydrofolate synthase Monofunctional of 105 kDa and 978 amino acids. This monofunctional enzyme consists of two main domains: an inactive N-terminal domain of methylene-THF dehydrogenase and cyclohydrolase from amino acid 31 to 348 and a larger C-terminal domain of formyl-THF synthetase (FTHFS) from amino acid 349 to 978. The G1691A variant is located in exon 16 of the gene and the R564H change is located in the C-terminal domain of the active formyl-THF synthetase (FTHFS).
	Structure of the MTHFD1L gene taken from the Ensembl platform.	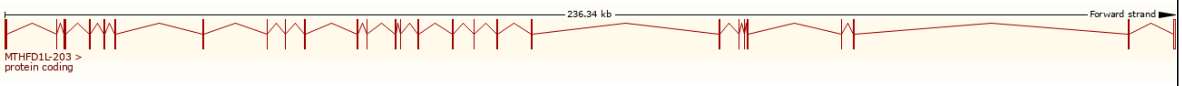
	Structure of the MTHFD1L protein taken from the Pfam platform.	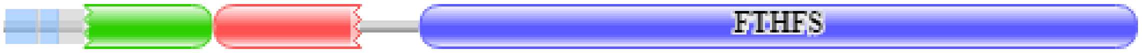
**APOE**<br>ID: 348	Apolipoprotein E<br>ID: P02649	The APOE gene is located in the 19q13.32 region, has a size of 3647 base pairs and is made up of 6 exons. This gene codes for apolipoprotein E of 36 kDa and 317 amino acids. These proteins contain multiple repeats of 22 residues that form an alpha helix pair (80-255), an LDL region, and other lipoprotein binding receptors (158-168), a heparin binding region (162-165), a region of Lipid binding and lipoprotein association (210-290), another heparin binding region (229-236), a Homooligomerization region (266-317) and a region of specificity for association with VLDL (278-290). The T388C variant is located in exon 4 of the gene and the C130R change is located in the tandem repeat region.
	Structure of the APOE gene taken from the Ensembl platform.	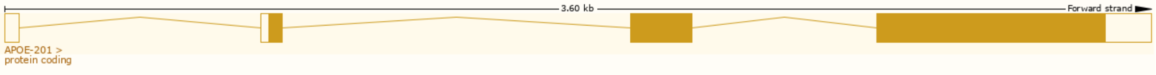
	Structure of the APOE protein taken from the Pfam platform.	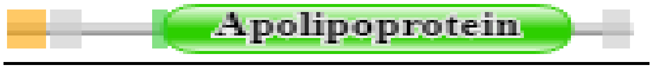

S8 Table. Structure of genes and proteins where candidate variants were identified in the family with Alzheimer's disease.
