# Supplementary material for: Mutations in SORL1 and MTHFDL1 possibly contribute to the development of Alzheimer’s disease in a multigenerational Colombian Family
Source: PLoS One. 2022 Jul 29;17(7):e0269955. doi: 10.1371/journal.pone.0269955 (PMC9337667; doi:10.1371/journal.pone.0269955)
Supplement: S9 Table — (PDF) [file pone.0269955.s018.pdf]

S9 Table. Energy and stereochemistry validation of structural models of SORL1 protein.

	Model	Software	QMEAN6	Z-score	Ramachandran plot		
					**Favored region**	**Allowed region**	**Outlier region**
SORL1_WT	Unrefined	**I-TASSER**	**0,3066**	**-10,0945**	**1056(70,9%)**	**288(19,3%)**	**145(9,7%)**
		PHYRE2	0,3140	-9,9349	1201(80.7%)	160(10,7%)	128(8,6%)
	Refined	FG-MD	0,3062	-10,1038	1040(69,8%)	315(21,2%)	134(9,0%)
		**Mod Refiner**	**0,3327**	**-9,5289**	**1149(77,2%)**	**249(16,7%)**	**91(6,1%)**
SORL1_R904W	Unrefined	**I-TASSER**	**0,2654**	**-10,9873**	**1009(67,8%)**	**326(27,9%)**	**154(10,3%)**
		PHYRE2	0,2063	-12,2694	1288(86.5%)	128(8.6%)	73(4.9%)
	Refined	FG-MD	0,2848	-10,5678	1037(69,6%)	309(20,8%)	143(9,6%)
		**Mod Refiner**	**0,3052**	**-10,1253**	**1046(77,0%)**	**246(16,5%)**	**97(6,5%)**

S9 Table. Energy and stereochemistry validation of structural models of SORL1 protein.
