# Supplementary material for: Mutations in SORL1 and MTHFDL1 possibly contribute to the development of Alzheimer’s disease in a multigenerational Colombian Family
Source: PLoS One. 2022 Jul 29;17(7):e0269955. doi: 10.1371/journal.pone.0269955 (PMC9337667; doi:10.1371/journal.pone.0269955)
Supplement: S10 Table — (PDF) [file pone.0269955.s019.pdf]

S10 Table. **Energy and stereochemistry validation of structural models of MTHFD1L protein.**

	Model	Software	QMEAN6	Z-score	Ramachandran plot		
					Favored region	Allowed region	Outlier region
MTHFD1L_WT	Unrefined	I-TASSER	0.273353	-11.383495	627(64.2%)	183(18.8%)	166(17.0%)
		**PHYRE2**	**0.462315**	**-7.045757**	**792(81.1%)**	**91(9.3%)**	**93(9.5%)**
	Refined	FG-MD	0.337980	-9.899940	685(70.2%)	151(15.5%)	140(13.3%)
		**Mod Refiner**	**0.483446**	**-6.560672**	**818(83.8%)**	**101(10.3%)**	**57(5.8%)**
MTHFD1L_R564H	Unrefined	I-TASSER	0.307382	-10.602347	631(64.7%)	197(20.2%)	148(15.2)
		**PHYRE2**	**0.492804**	**-6.345860**	**769(78.8%)**	**116(11.9%**	**91(9.3%)**
	Refined	FG-MD	0.519764	-5.726972	790(80.9%)	113(11.6%)	73(7.5%)
		**Mod Refiner**	**0.554845**	**-4.921662**	**841(86.3%)**	**89(9.1%)**	**44(4.5%)**

S10 Table. **Energy and stereochemistry validation of structural models of MTHFD1L protein.**
