# Supplementary material for: Mutations in SORL1 and MTHFDL1 possibly contribute to the development of Alzheimer’s disease in a multigenerational Colombian Family
Source: PLoS One. 2022 Jul 29;17(7):e0269955. doi: 10.1371/journal.pone.0269955 (PMC9337667; doi:10.1371/journal.pone.0269955)
Supplement: S11 Table — (PDF) [file pone.0269955.s020.pdf]

## S11 Table. Primer information for Sanger sequencing.

| GEN | VARIANT | Primers | | Tamaño (pb) |
|---|---|---|---|---|
| SORL1 | R904W | F: | ATGCTGAAATAACCAGCCGGA | 421 |
| | | R: | CTCATATGCTCCTCACCCGCT | |
| MAPT | R556P | F: | CTCGAGTCCTGGCTTCACTC | 417 |
| | | R: | CCACGACTCCACGCTCAAC | |
| CHAT | R257Q | F: | CTCAGGACACTCTGGCTGAC | 491 |
| | | R: | AGCAGAAACCAGTGAGTGCG | |
| ABCA7 | A877T | F: | CTCTGTGAAGGGGGCTACTC | 725 |
| | | R: | CAGAATCCCCAGCTCTTACCT | |
| MTHFD1L | R564H | F: | TCTGAGCAGCGTACATGGTG | 1498 |
| | | R: | GGCTGGGAAGATCACTGGAC | |
| APOE | C130R | F: | CCCAGGAACTGAGGTGAGTG | 1499 |
| | | R: | ACTGAGGCTGGGGCTTAGAG | |
| APOE | C176R | F: | CCCAGGAACTGAGGTGAGTG | 1499 |
| | | R: | ACTGAGGCTGGGGCTTAGAG | |

**S11 Table. Primer information for Sanger sequencing.**
