# Supplementary material for: Mutations in SORL1 and MTHFDL1 possibly contribute to the development of Alzheimer’s disease in a multigenerational Colombian Family
Source: PLoS One. 2022 Jul 29;17(7):e0269955. doi: 10.1371/journal.pone.0269955 (PMC9337667; doi:10.1371/journal.pone.0269955)
Supplement: S1 Appendix — (PDF) [file pone.0269955.s021.pdf]

S1 Appendix. Clinical evaluation of Index case (III:5).

The patient of 67 years old during evaluation, reports a chief complaint of short-term memory impairment (in episodic and semantic memory) and unimpaired long-term memory, with onset of symptoms at 63 years old and a slowly progressive course. He had additional neuropsychiatric symptoms as insomnia, distractibility, depressive symptoms, and aggressive behavior. Two years after AD onset, he suffered a mild traumatic brain injury (TBI) that accentuated his clinical state. At 65 years old, he suffered a convulsive status epilepticus (CSE) and he was hospitalized, where they performed a cerebral and lung computed tomography (CT) showing lung cancer with cerebral metastasis. During this hospitalization he was independent for basic activities of daily living. He continued having seizures during night, with generalized rigidity and upward gaze deviation. In the same year, he began to suffer slurred speech, left hemiparesis, diplopia, and visual hallucinations (microzoopsias) in a progressive matter. At 67 years old, his clinical status progressed, requiring help for basic daily activities. In the last physical examination, we found the patient in a wheelchair unable to walk and talk (severely anomic and hypospontaneity), with moderate apathy, with generalized muscular atrophy, left papilledema, right eye ptosis, apparently oculomotor palsy, quadriparesis, and right myoclonus. Results of an MRI showed, lesions related to metastatic neoplastic disease and microangiopathy. We did not perform neuropsychological testing as he was unable to perform the tests. He died at 67 years of age, and we performed a research autopsy with brain extraction and processing according to Neuroscience Research Group brain processing protocol. We also obtained histopathological smears according to CERAD protocol stained with hematoxylin-eosin and immunohistochemistry. There was a family history of neurodegenerative disease, HTN, hypoacusis in five siblings, and lung cancer in three siblings. We conclude the patient had a pattern of disease compatible with EOAD, but with increased neurologic motor deterioration associated with the metastatic lung cancer. Gross Pathology: Weight of the encephalon: 1035.3 grs. Weight of the posterior fossa: 142.3 grs. Meninges of normal appearance; cerebrospinal fluid of hematic appearance. Brain with mild to moderate global atrophy is observed, multiple yellow spots are observed, with poorly defined limits and dimensions that can vary from 0.5 centimeters to 4 centimeters in diameter) that compromise various brain regions (right basal portion, in relation to the olfactory bulb; in the right middle frontal gyrus, premotor area; in the upper lobe of the right parietal; in the right occipital region; in the left upper parietal lobule; in the left

occipital lobe; in the lower portion of the left precentral gyrus. Slightly reduced uncus of size; on palpation the left uncus is more hardened than the right one.
