# Supplementary material for: Mutations in SORL1 and MTHFDL1 possibly contribute to the development of Alzheimer’s disease in a multigenerational Colombian Family
Source: PLoS One. 2022 Jul 29;17(7):e0269955. doi: 10.1371/journal.pone.0269955 (PMC9337667; doi:10.1371/journal.pone.0269955)
Supplement: S2 Appendix — (PDF) [file pone.0269955.s022.pdf]

S2 Appendix. Clinical evaluation of Affected family member (III:10).

The patient of 57 years old during the first evaluation, with remarkable past medical history of neonatal late meningitis with visual impairment in left eye as sequel, typhoid fever at 15 years old, chronic obstructive pulmonary disease (COPD), HTN-Dyslipidemia since 48 years old, pneumonia in three occasions requiring intensive care unit (ICU) and endotracheal intubation in two occasions, moderate TBI at 37 years old without sequels, B12 deficiency and depression at 56 years old. Also, a family history of cancer, HTN and AD. He chief medical complaint is short-term memory impairment since first symptom onset at 55 years old, with slow progression and not spatially disoriented. In the physical examination, he had difficulty performing the tandem walking test, was unable to stand in a single foot for more than 10 seconds, with everything else normal in the neurological and general physical examination. Neuropsychological testing: MMSE: Time Orientation: 4/5, Place Orientation: 5/5, Fixation Memory: 3/3, Attention and Calculation: 5/5, Evocation Memory: 1/3, Language: 9/9, Total: 27. Verbal fluency: 18, Denomination Abbreviated Boston Test: 15/15, TMT A: 24 right. 0 wrong. 52 seconds, MIS: Free recall 4, Categorical recall 2. Total points 10. WAIS-III: 30 of 133. Memory Capacity Test (Buschke): Free recall 13 with 1 intrusion. Recall with clues list one 16, list two 9, intrusions list one 0, intrusions list two 1 and 3 contaminations. Recall clue of the two lists, list one 14, list two 11, total 27, intrusions list one 0, intrusions list two 1. The scale of memory disorders: QP 16. 17. Yesavage depression scale: 3/15. Normal. Zung depression scale: 47. Mild depression. FAST: 2/16. Normal. EDG: 2/7. Normal. Katz: 0/6. Independent for basic activities. Lawton and Brody: 8/8. Independent. Brain CT with hypodensity in white substance, related to microangiopathic leukoencephalopathy. Based on these findings the diagnosis was EOAD.
